# A new, rare, small-ranged, and endangered mountain snake of the genus *Elaphe* from the Southern Levant

**DOI:** 10.1038/s41598-023-30878-4

**Published:** 2023-03-24

**Authors:** Daniel Jablonski, Marco Antônio Ribeiro-Júnior, Evgeniy Simonov, Katarína Šoltys, Shai Meiri

**Affiliations:** 1 Department of Zoology, Comenius University in Bratislava, Bratislava, Slovakia; 2 The School of Zoology and The Steinhardt Museum of Natural History, Tel Aviv University, Tel Aviv, Israel; 3 Severtsov Institute of Ecology and Evolution, Russian Academy of Sciences, Moscow, Russia; 4 Department of Microbiology and Virology, Comenius University in Bratislava, Bratislava, Slovakia; 5 Comenius University Science Park, Bratislava, Slovakia

**Keywords:** Evolution, Taxonomy, Zoology, Herpetology, Biodiversity, Biogeography, Molecular ecology

## Abstract

The genus *Elaphe* Fitzinger, 1833 includes 17 species of charismatic, large-sized, non-venomous, Eurasian snakes. In the Western Palearctic, the genus is represented by three species from the *Elaphe quatuorlineata* group ranging from the Apennine peninsula to Central Asia. The southernmost population of this group is distributed in the mountains of the Southern Levant, with more than 400 km gap to other *Elaphe* populations. This population has been known to science for only 50 years and is virtually unstudied due to its extreme rarity. We studied these snakes’ morphological and genetic variation from the three countries where they are known to occur, i.e., Israel (Hermon, the Israeli-controlled Golan Heights), Lebanon, and Syria. We used nine mitochondrial and nuclear genes, complete mitogenome sequences, and a comprehensive morphological examination including published data, our own field observations, and museum specimens, to study its relationship to other species in the group. The three currently recognized species of the group (*E*. *quatuorlineata*, *E*. *sauromates*, *E*. *urartica*), and the Levant population, form four deeply divergent, strongly supported clades. Three of these clades correspond to the abovementioned species while the Southern Levant clade, which is genetically and morphologically distinct from all named congeners, is described here as a new species, *Elaphe druzei* sp. nov. The basal divergence of this group is estimated to be the Late Miocene with subsequent radiation from 5.1 to 3.9 Mya. The revealed biogeography of the *E*. *quatuorlineata* group supports the importance of the Levant as a major center of endemism and diversity of biota in Eurasia. The new species is large-sized and is one of the rarest snakes in the Western Palearctic. Because of its small mountain distribution range, in an area affected by land use and climate change, the new *Elaphe* urgently needs strict protection. Despite political issues, we hope this will be based on the cooperation of all countries where the new species occurs.

## Introduction

The old-world rat snakes in the genus *Elaphe* Fitzinger, 1833 comprise 17 species, including some of the most charismatic, large-sized, non-venomous snakes of the Palearctic and Oriental zoogeographic realms^1–4^. Since ancient times, rat snakes featured in human culture, myths, medicine, and religion. Especially three species of rat snakes, *E*. *quaturorlineata* (Bonnaterre, 1773), *Zamenis longissimus* (Laurenti, 1768) (formerly *E*. *longissima*), and *Z. situla* (Linnaeus, 1758) (formerly *Elaphe situla*), were believed protect people from ailments and venomous snakebites^5^. Such snake cults are an old phenomenon with origins at least as early as ancient Egyptian, Jewish^6^, (Numbers 21: 9), and Mesopotamian cultures, and perhaps as early as in the Natufian period some 12,000 years ago, as well as prevailed later in ancient Greece and Rome^7–9^. Today, such traditions still exist, for example in the Abruzzo area of Italy (Fig. S15), and in Greece^10^. The snakes of the genus *Elaphe* and allied taxa, ranked today in other genera (e.g., *Coelognathus*, *Gonyosoma*, *Zamenis*), are also favourite species in herpetoculture and due to their large size, attractiveness, calm nature, and easy breeding in captivity^1^. They are also often studied and thus form one of the best-known genera of snakes worldwide (e.g.^1,11^). This is, however, not true for the southernmost population of *Elaphe* in the Western Palearctic occurring in the mountains of the Southern Levant.

The first well-verified record of the *Elaphe quatuorlineata* group from the Levant was presented by Hermann Zinner^12^ half a century ago, five years after the Israeli occupation of the Golan Heights and Mt. Hermon in 1967. In June 1971 Mr. Yossi Levari, the curator of Beit Ussishkin Natural History Museum, collected a freshly killed large (1300 mm) female snake, a few kilometres from Majdal Shams, on the southern slope of Mt. Hermon (33.279°N, 35.776°E, around 1300 m a.s.l.)^12^. It was catalogued at the Beit Ussishkin Museum in Kibbutz Dan and is now deposited at the Steinhardt Museum of Natural History, Tel Aviv University, Tel Aviv, Israel (TAU; voucher number TAU-R 19438; Fig. S7). Zinner^12^ morphologically compared the specimen with the description of *E*. (*quaturolineata*) *sauromates* (Pallas, 1814). He stated that the specimen differs from more northerly distributed subspecies by a very dark coloration and strongly keeled dorsal scales. He has been surprised why such a large species of snake was not previously observed “between southwestern Turkey and the Lebanon”. He explained this by the cryptic behaviour of these snakes and suggested that the species must be present also in other regions of the Levant. Biton et al.^13^ presented fossil records of a snake resembling members of *E*. *quatuorlineata* group from the Pleistocene, in the Hula Valley, Israel. Lev et al.^14^ likewise identified the remains of such a snake from the Natufian period of El-Wad Terrace, Mt. Carmel, Israel. After 1971, more field observations, from the Israeli-controlled territory of Golan Heights, SW Syria, and SE Lebanon followed^15–21^ but generally, these snakes are rarely observed, not studied, presumably threatened, and represent one of the most enigmatic snake populations in the Western Palearctic.

Much has changed since Zinner’s publication^12^ in our understanding of the distribution, biogeography, and evolution of the genus *Elaphe*, particularly in the *E*. *quatuorlineata* group. First, molecular-phylogenetic, and taxonomic approaches showed that *E*. *sauromates* (Pallas, 1814) has a long independent evolutionary history (from *E*. *quatuorlineata*, of which it was considered a subspecies), which is also suggested by its morphology and distribution. It is a valid species as confirmed by studying the genetic diversity and morphology of the group^22–28^. The two species had different centres of their initial divergence (probably in the Balkans and in western Anatolia, respectively). More surprisingly, later studies showed much genetic diversity between eastern and western populations of "*E. sauromates*", suggesting the existence of another species^23,25^. This eastern population was recently described as *E*. *urartica* Jablonski, Kukushkin, Avcı, Bunyatova, Ilgaz, Tuniyev, Jandzik, 2019. *Elaphe urartica* is, so far, known from eastern Anatolia, the Transcaucasian region, and Dagestan. It is a cryptic species moderately morphologically differentiated from its sister species *E*. *sauromates* and genetically well defined. There is no current evidence that *E*. *sauromates* and *E*. *urartica* are sympatric anywhere (e.g., in Anatolia). Jablonski et al.^25^ predicted that the contact zone could follow the so-called Anatolian diagonal (central part of Anatolia) something that was observed in many reptile species in the region. They also suggested that the southernmost populations of *E. quatuorlineata* group in the Levant could be affiliated with *E*. *urartica.* This was not tested, and the Levant population is currently assigned to *E*. *sauromates*^12,22,29,30^. On the other hand, a high level of reptile endemism in Southern Levant region^31^, and the wide (~ 400 km) gap between the Southern Levant *Elaphe* population and closest populations of the genus, may imply the Levant is a fourth geographic centre of independent evolution in the *E*. *quatuorlineata* group and the population potentially deserving species status.

To further investigate the phylogenetic relationships, biogeographic origin, level of divergence, and taxonomic status of this rare and enigmatic population from the mountains of the Southern Levant, we studied genetic (both matrilinear and bi-parental DNA markers), morphological (measurements, pholidosis, body scalation, color pattern, and hemipenes), and ecological data of these snakes and compared them with known species of the genus *Elaphe*.

## Results

### Genetics

#### Molecular phylogeny and genetic diversity

**Figure 1:**
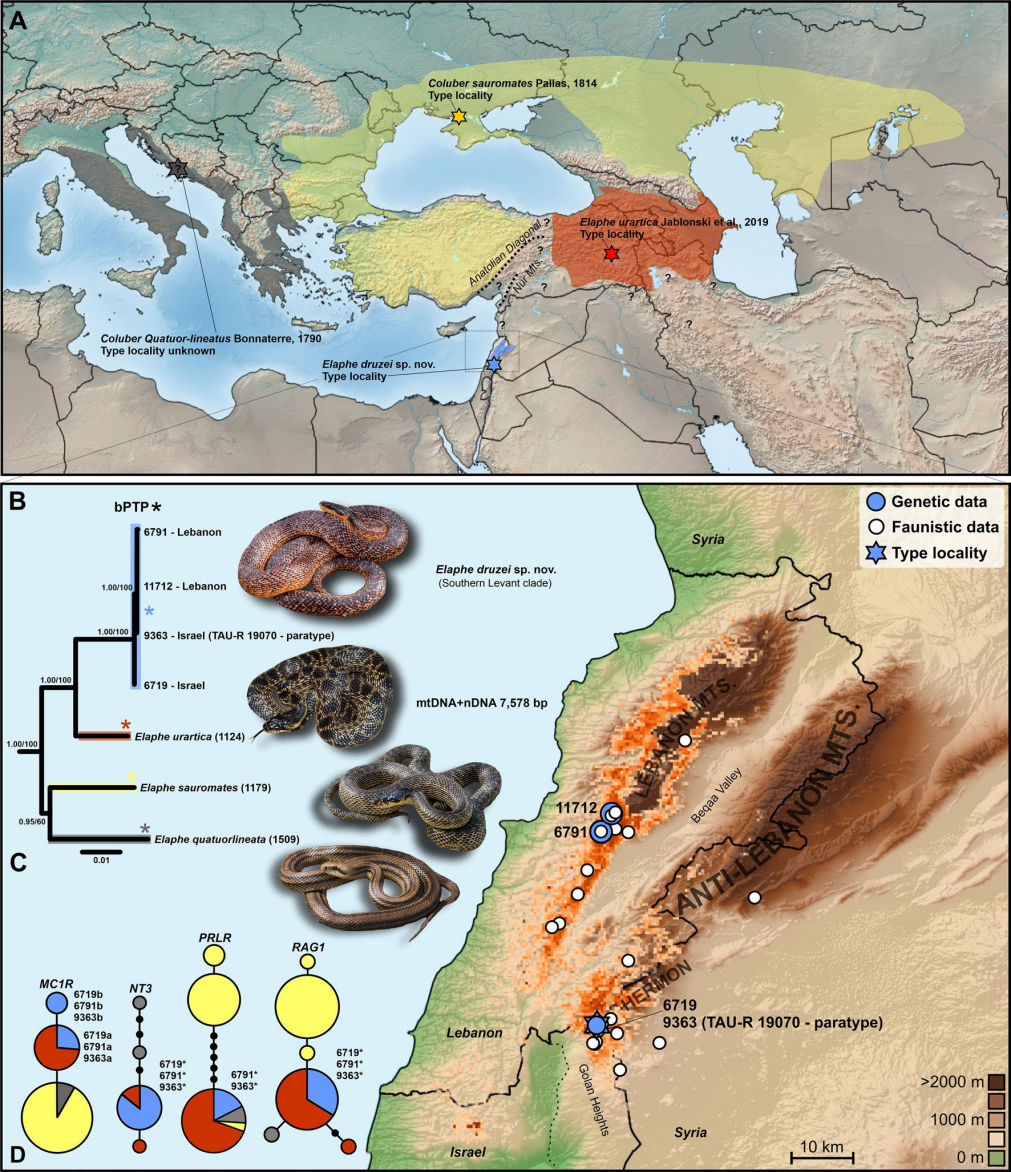
(**A**) The current distribution of western Palearctic *Elaphe* species from the *E*. *quatuorlineata* group with their type localities: *E*. *quatuorlineata* (grey), *E*. *sauromates* (yellow), *E*. *urartica* (red), *E*. *druzei* sp. nov. (blue), according to Sindaco et al. ^29^, Jablonski et al. ^25^ and this study. Question marks indicate places of unclear or possible occurrence. (**B**) The map of the Southern Levant with distribution points of *Elaphe druzei* sp. nov. (white circles) used for species distribution model (orange layer) and localities used for genetic analyses (blue circles; for details see Table S1). (**C**) The phylogenetic hypothesis and relationships of the *E*. *quatuorlineata* group based on the concatenated dataset of mitochondrial and nuclear DNA supplemented by the species delimitation analysis (bPTP) supporting recognized clades (*). The numbers above the branches of the tree represent Bayesian posterior probabilities/ bootstraps support values. (**D**) Nuclear allele networks of the phased sequences of *MC1R*, *NT3*, *PRLR* and *RAG1* (Table S2) of the *E*. *quatuorlineata* group. Species colours follow those used in the map (**A**) and the tree (**C**). Circle sizes are proportional to the number of alleles. A small black circle indicates a missing or hypothetical allele. Different alleles of a single heterozygous specimen are coded as a and b, while an asterisk indicates an allele of a homozygous specimen. The code numbers are the same as those used in Tables S1 and S2. Inset photographs: Daniel Jablonski, Mark Pestov (*E*. *sauromates*), and Ilya S. Korshunov (*E*. *urartica*). The map was generated using QGIS 3.28 available at https://qgis.org/.

**Figure 2:**
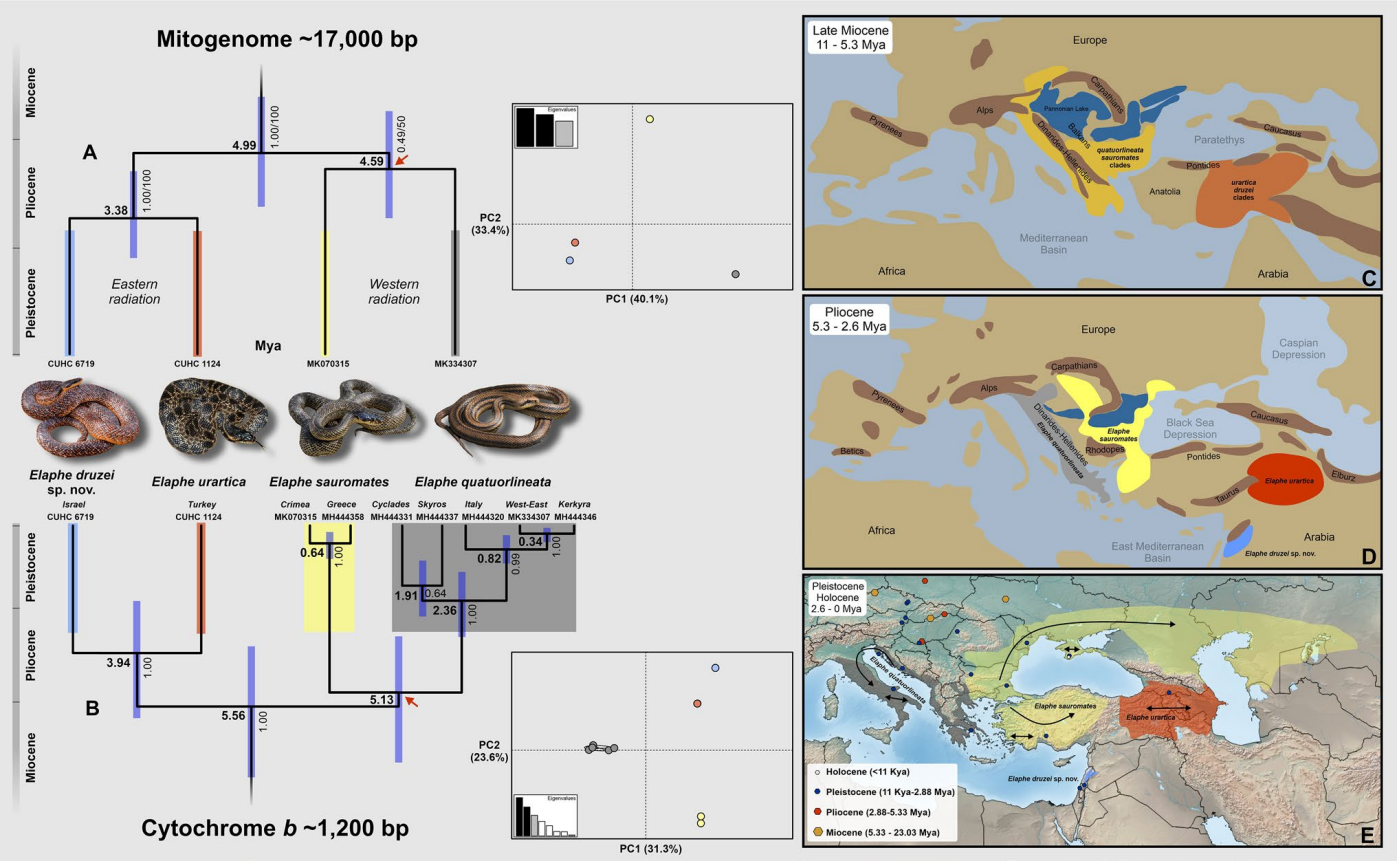
The dated phylogeny and Principal Component Analysis (PCA) based on full mitogenome sequences (**A**) in comparison with the Cyt *b* dataset (**B**) of the *Elaphe quatuorlineata* group, showing the studied species. Colours on branches and in graphs follow those used in Fig. 1. Numbers between clades of species stands for the estimated time of divergence (Mya: in millions of years ago; mean and 95% HPD). The numbers with branches represent Bayesian posterior probabilities/bootstraps support values taken from independent IQ-TREE and MrBayes analyses (mitogenome) or from BEAST analysis (Cyt *b*). The red arrows indicate constrained nodes. The simplified hypothesis on the historical biogeography (maps modified according to Popov et al.^32^) suggested for the group based on the molecular dating (**A**, **B**): the Late Miocene (**C**), Pliocene (**D**) and the late Pleistocene and Holocene (**E**) with fossil records for the genus *Elaphe* (the fosFARbase database and Schulz^1^), representing the main geological periods (**E**). Arrows display the approximate routes of post-glacial colonization according to the distribution of the mitochondrial diversity and structure (Jablonski et al.^25^; this study). Insets photographs: Daniel Jablonski, Mark Pestov (*E*. *sauromates*) and Ilya S. Korshunov (*E*. *urartica*).

The final concatenated dataset of 7578 bp comprised sequences of four mitochondrial (mtDNA) genes and five nuclear genes. This dataset is formed by three sequence chains of currently recognized taxa (*E*. *quatuorlineata*, *E*. *sauromates*, *E*. *urartica*; Fig. 1A) of the *E*. *quatuorlineata* group, four sequence chains originating from *Elaphe* populations distributed in the Southern Levant (Table S1, Fig. 1B), and 16 sequences chains of outgroup taxa representing the genera *Elaphe, Euprepiophis,* and *Gonyosoma* (Table S2). In both phylogenetic analyses (ML & BI; Figs. 1C, and S1, S2) of the concatenated dataset, the *E*. *quatuorlineata* group was divided into four deeply divergent clades (60–100 bootstrap/0.95–1.00 posterior probabilities). Three clades correspond to the currently recognized species of this group while the fourth (Southern Levant clade), represented by snakes from mountains of the Israeli-controlled Golan Heights (CUHC 6719, 9363) and Lebanon (CUHC 6791, 11712), is described below as a new species. The results suggest two main sister radiations in the evolution of the group, the first (Western) including *E*. *quatuorlineata* (currently distributed in the western Balkans, and the Apennine peninsula), and *E*. *sauromates* (eastern Balkans, East European Plains, western part of Central Asia). The second (Eastern) includes *E*. *urartica* (eastern Turkey, Transcaucasian region) and the new clade from the Southern Levant (Fig. 2). The new taxon is a sister species of *E*. *urartica*, supporting a hypothesis of an Eastern geographic origin of both clades (Fig. 1A,C).

The same topology of the *E*. *quatuorlineata* group was obtained from full mitogenome sequences (~ 17,000 bp; Fig. 2A) in both ML and BI analyses. Lower bootstrap and posterior probability supports were detected in the relationship between mitogenomes of *E*. *quatuorlineata* and *E*. *sauromates* (50 and 0.49, respectively), similarly as in the analysis of the concatenated dataset (60). Given the results of analyses we consider the phylogenetic hypothesis obtained from the concatanated analysis as more reliable.

The network analysis of available nuclear sequences of the *E*. *quatuorlineata* group (genes *MC1R*, *NT3*, *PRLR*, *RAG1*) divided datasets into three to six alleles indicating low levels of sequence evolution or incomplete lineage sorting in the four detected clades (Fig. 1D). *Elaphe quatuorlineata* and *E*. *sauromates* share one *MC1R* allele. A second allele is shared by *E*. *urartica*, and the Southern Levant clade. A third allele is exclusive to the Southern Levant clade. In *NT3*, one allele is exclusive for *E*. *urartica*, a second allele is shared by *E*. *urartica*, and the Southern Levant clade, and two alleles are exclusive to *E*. *quatuorlineata*. In the *PRLR* dataset sequences of all four clades share one allele but *E*. *sauromates* also has two, deeply divergent, exclusive alleles. In *RAG1*, *E*. *quatuorlineata* and *E*. *sauromates* exclusively share separate alleles while *E*. *urartica* is represented by two alleles one of which is shared with the Southern Levant clade. This is consistent with phylogenetic relationships based on the concatenated and mitochondrial datasets and the geographic origin of clades.

In accordance with the phylogenetic analyses, the PCAs revealed four distinct clusters corresponding to the currently recognized species of the *E*. *quatuorlineata* group and the new clade from the Southern Levant with non-overlapping 95% confidence intervals (Fig. 2A,B). The first (PC1) and second (PC2) principal components explain 40.1% and 33.4% of the observed variance in the mitogenome dataset, and 31.3% and 23.6% in Cyt *b* dataset.

According to divergence dating analysis (Figs. 2 and S3, S4), the basal split of the Western and Eastern radiation of the group was estimated at 4.99 (6.42–3.70 of 95% HPD) Mya based on the full mitogenomes, and 5.56 (7.59–3.64) Mya based on Cyt *b* only (both Late Miocene). The split between *E*. *quatuorlineata* and *E*. *sauromates* occurred 4.59 (5.97–3.35) Mya (at the border between Miocene and Pliocene), and *E*. *urartica* diverged from the Southern Levant clade 3.38 (4.52–2.39) Mya (in the Pliocene). Similar but slightly older dates were inferred from the Cyt *b* dataset with the divergence between *E*. *quatuorlineata* and *E*. *sauromates* estimated on 5.13 (7.15–3.34) Mya (Late Miocene), with subsequent progressive diversification of *E*. *quatuorlineata* lineages (sensu^28^) from 2.26 to 0.34 (3.35–0.34) Mya (Pliocene/Pleistocene). The split between *E*. *urartica* and the Southern Levant clade was estimated using Cyt *b* at 3.94 (5.81–2.27) Mya (Pliocene) (Figs. 2 and S3, S4).

The analysed protein-coding mtDNA of the Southern Levant population from Mt. Hermon (Anti-Lebanon Mountains) and the Lebanon Mountains, contains very low genetic diversity; *COI* (n = 3; 872 bp): three haplotypes with π = 0.24%; *ND4* (n = 4; 882 bp): one haplotype, π = 0%; Cyt *b* (n = 2; 1119 bp): two haplotypes with π = 0.18%.

The complete mitochondrial genomes of the sequence from the Levant population (CUHC 6719) and *E*. *urartica* (CUHC 1124) are 17,182 and 17,184 bp long, respectively (GenBank accession numbers: OP613266 and OP613267). They contain 13 protein-coding genes (PCGs), two rRNA genes, 22 tRNA genes, and two control regions. The overall base composition of the mitogenomes in descending order was 34.7/34.6%—A, 26.9/26.9%—C, 25.6/25.7%—T, 12.8/12.8%—G, with an equal A + T bias (60.3%). This composition is almost identical to the published mitogenomes of *E*. *quatuorlineata* and *E*. *sauromates*^26,34^.

The uncorrected *p* distances between all clades and markers were high (Table 1). The Southern Levant clade differs from other clades by 1.92% (*E*. *urartica*) to 3.52% (*E*. *quatuorlineata*) in *16S*, from 4.80% (*E*. *urartica*) to 7.33% (*E*. *quatuorlineata*, *E*. *sauromates*) in *COI*, 4.80% (*E*. *urartica*) to 7.43% (*E*. *sauromates*) in *ND4*, and 5.27% (*E*. *urartica*) to 7.32% (*E*. *quatuorlineata*) in Cyt *b*. For the complete mitogenome sequences, we detected values from 4.20% to 6.10% that differentiated the new clade from other congeners of the group.

**Table 1:** Uncorrected *p* distances (%) between investigated sequences and markers of the *Elaphe quatuorlineata* group.

*16S*/*COI*/*ND4*/*Cyt* b/mitogenome (%)	*E. quatuorlineata*	*E*. *sauromates*	*E*. *urartica*	*E*. *druzei* sp. nov
*E. quatuorlineata*	–			
*E*. *sauromates*	2.29/7.80/7.20/6.70/5.57	–		
*E*. *urartica*	3.82/8.95/5.49/7.06/6.04	2.98/7.45/6.17/6.07/5.22	–	
*E*. *druzei* sp. nov	3.52/7.33/6.74/7.32/6.10	3.22/7.33/7.43/6.52/5.46	1.92/4.80/4.80/5.27/4.20	–

The bPTP species delimitation analysis of the concatenated dataset of 23 sequence chains (representing by 20 taxa), recognized 19 putative species with high statistical support (0.99–1.00). It clearly supported the hypothesis of four clades in the *E*. *quatuorlineata* group, each representing a different species (Fig. 1C).

#### Morphology

**Figure 3:**
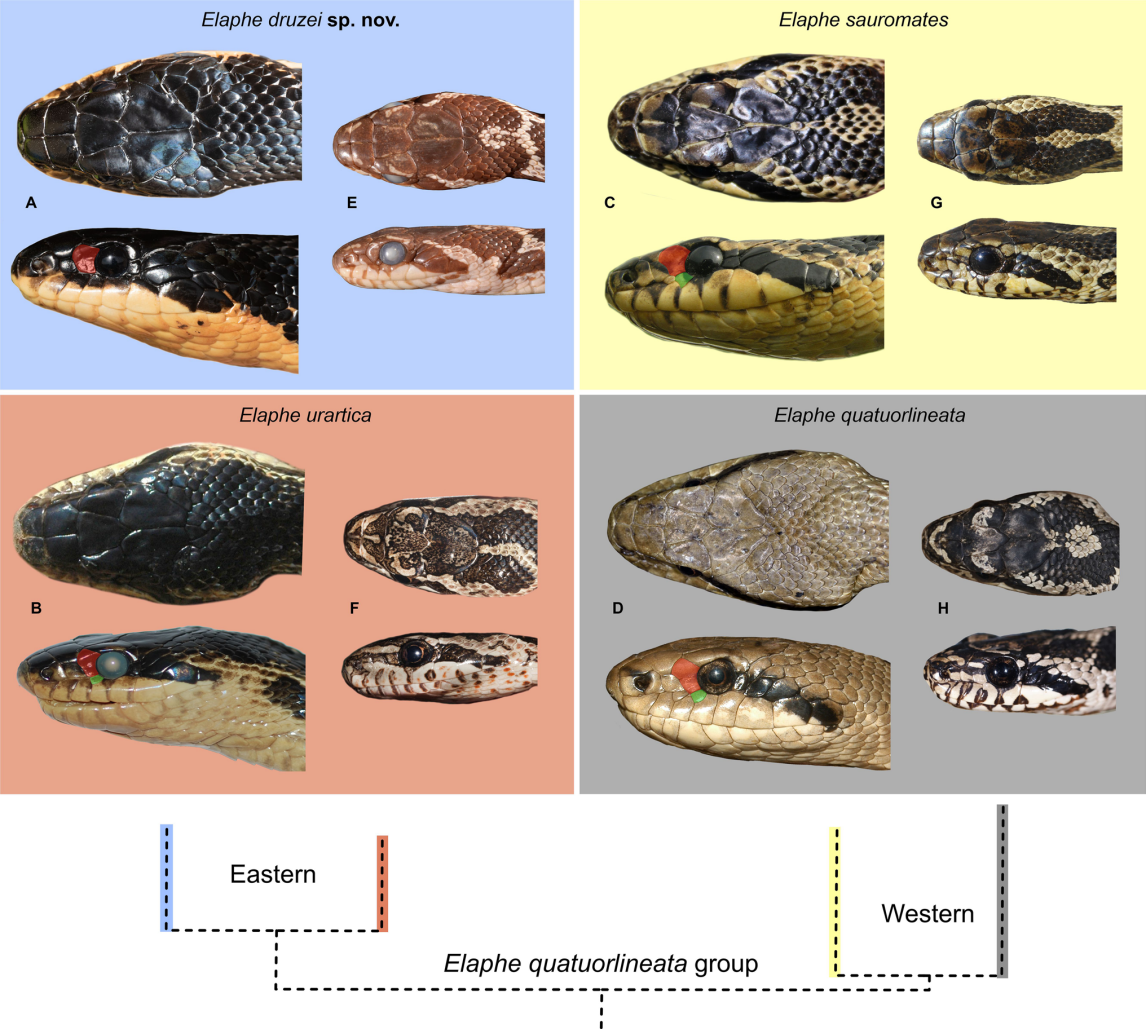
The comparison of preocular scales (red upper, green lower), head pattern and coloration in adult (**A**–**D**) and juvenile (**E**–**H**) stages of *Elaphe quatuorlineata* group arranged along the schematic phylogeny: *Elaphe druzei* sp. nov. from Lebanon (adult) and Israel (juvenile), *E*. *urartica*, Turkey (holotype ZDEU 26/2012) and Armenia (juvenile), *E*. *sauromates*, Ukraine, and *E*. *quatuorlineata*, Croatia and Italy. Photographs by Daniel Jablonski, Marco Antônio Ribeiro-Júnior and Matteo Di Nicola (**A**, **D**, **E**, **H**), Aziz Avcı (**B**), Ilya S. Korshunov (**F**), and Oleg V. Kukushkin (**C**, **G**).

**Figure 4:**
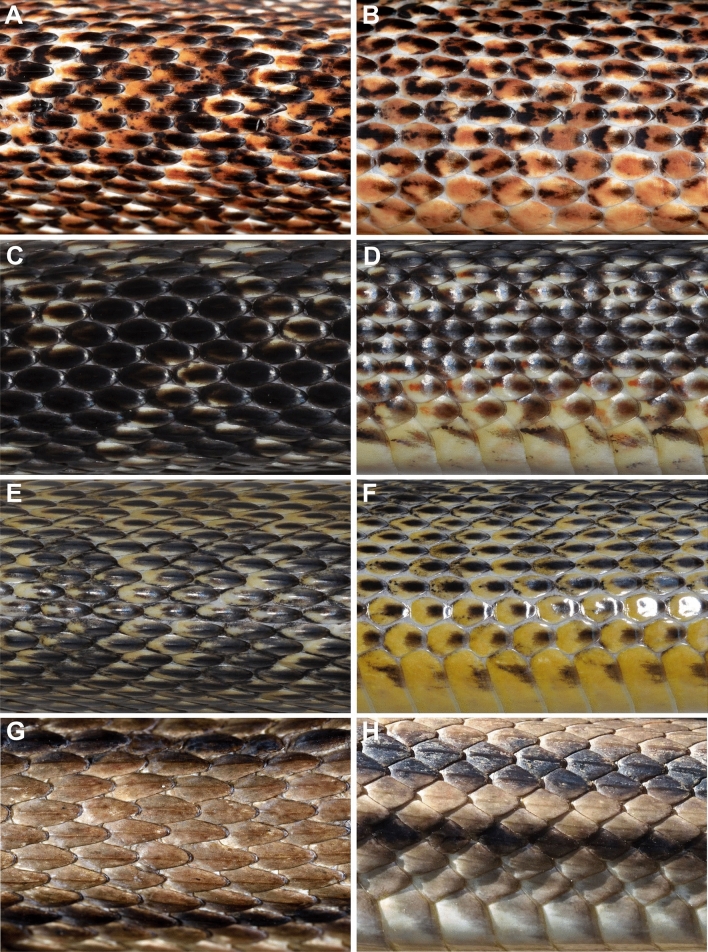
The comparison of dorsal and lateral scalation and its shape and keeling of the *Elaphe quatuorlineata* group. (A, B) *E*. *druzei* sp. nov. (Lebanon, photographs by D. Jablonski), (C, D) *E*. *urartica* (Armenia, photographs by Roman A. Terentev), (E, F) *E*. *sauromates* (Ukraine, photograps by Roman A. Terentev), (G, H) *E*. *quatuorlineata* (Italy, photographs by Matteo Di Nicola).

**Figure 5:**
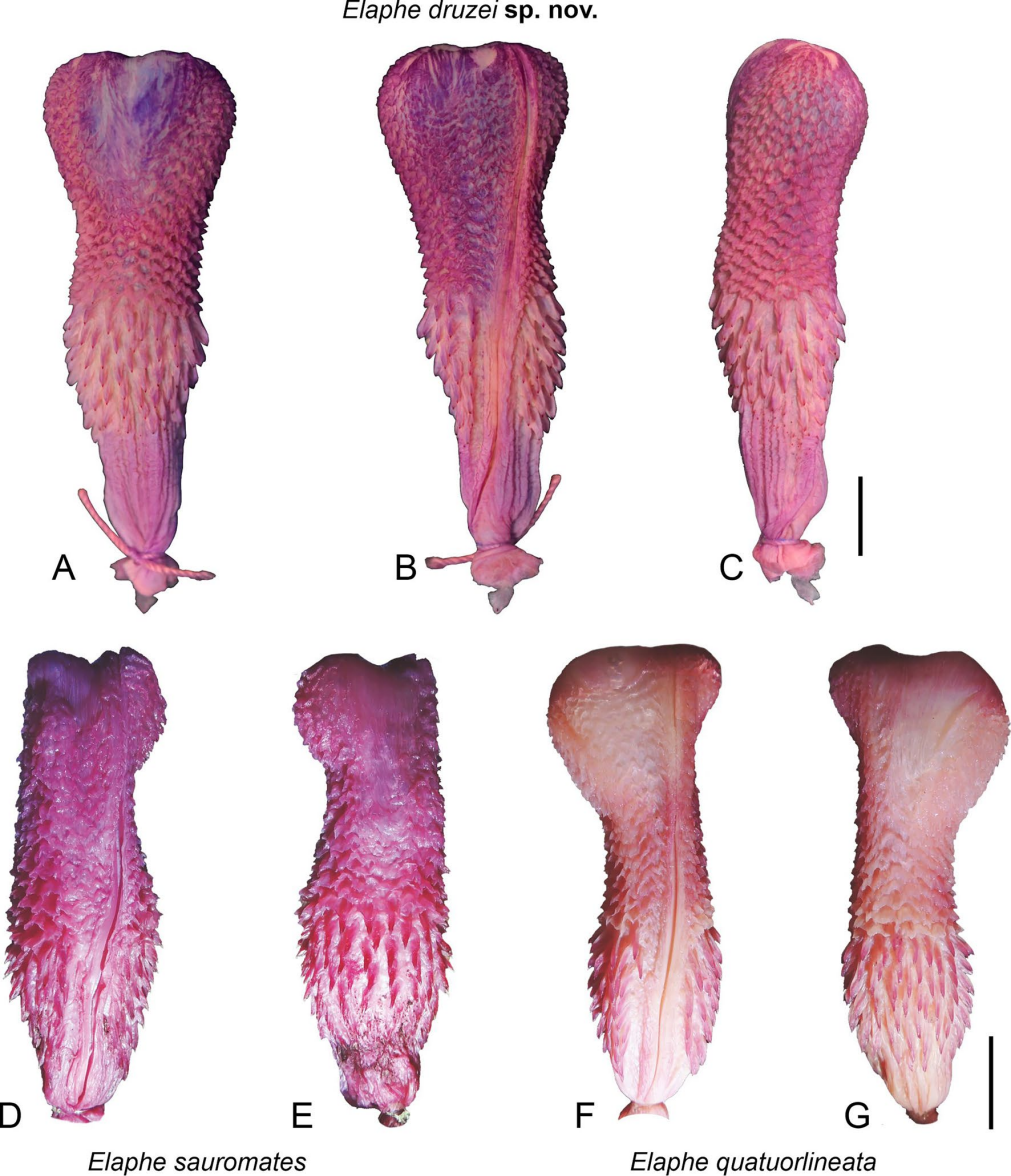
Asulcate (**A**), sulcate (**B**), and lateral faces (**C**) of the hemipenis of *Elaphe druzei* sp. nov. (TAU-R 13149), compared to *E*. *sauromates* (**D**, **E**) and *E*. *quatuorlineata* (**F**, **G**) both from Bulgaria (courtesy of Kostadin Andonov) (**D** and **F**: sucale faces; **E** and **G**: asulcate faces). Scale bar = 1 cm.

We morphologically examined specimens belonging to the Southern Levant clade (Tables 2, 3) and compared them with all other *Elaphe* species, mainly *E*. *sauromates* and *E*. *urartica,* in which the Levantine population was formerly ranked. Quantitative differences in dorsalia at forebody, midbody, and hind body, ventralia, subcaudalia, sublabialia, preoculare, and snout–vent length, allowed us to distinguish the new taxon from nine other species of the genus (Table 4). Four qualitative characters permitted us to distinguish it from all remaining species: 1. the dorsal surfaces of head, neck, and anterior-most part of the body are homogeneously black; 2. the lateral surface of the head, above the supralabialia is homogeneously black (Fig. 3), and the dorsal surface of the body is yellowish with wide, oval black-brown blotches surrounded by black rings in adults; 3. the ornamentation of scales on the dorsal surface of the body (dorsal body scales distinctly keeled and elongated; dorsolateral scales on the body are slightly elongated, rather smooth; Fig. 4); 4. the ornamentation of the hemipenis (the base is ornamented by long spines), is formed by spinulated calyces at about ½ of the total organ length, and the apical area and the body of the hemipenis has several, conspicuous spinulated calyces; Fig. 5). The new clade is not conspecific with any previously recognized congener, and therefore, based on the overall congruence with species delimited in the molecular analyses, and morphological distinctiveness, we propose its taxonomic reassessment.

**Table 2:** Summary of the variation of meristic characters and measurements (mm) in *Elaphe druzei* sp. nov. (own data), *E. sauromates* and *E. urartica* (Jablonski et al.^25^ and Jablonski pers. data).

Meristic characters	*Elaphe druzei* sp. nov	*E. sauromates*	*E. urartica*
	(n = 22)	(n = 63)	(n = 32)
Preoculare	1–2	1–3	1–3
	(1.35 ± 0.49)	(1.89 ± 0.52)	(1.52 ± 0.59)
Postoculare	2–3	1–3	1–3
	(2.06 ± 0.24)	(2 ± 0.17)	(2.03 ± 0.25)
Temporale	2–4	1–3	2–3
	(2.35 ± 0.49)	(2 ± 0.17)	(2.46 ± 0.63)
Posttemporale	2–4	2–5	2–4
	(3.47 ± 0.71)	(3.40 ± 1.02)	(3.58 ± 0.61)
Supralabialia	8–9	7–10	8–9
	(8.06 ± 0.24)	(8.16 ± 0.42)	(8.02 ± 0.13)
Supralabialia contacting the eye	4th and 5th (n = 12)	4th and 5th (n = 34)	4th and 5th (n = 13)
	4th and 5th, and 5th and 6th (n = 2)	4th and 5th, and 5th and 6th (n = 4)	4th and 5th, and 5th and 6th (n = 1)
		5th and 6th (n = 1)	
		5th and 6th, and 6th and 7th (n = 1)	
		5th and 6th, and 4th, 5th and 6th (n = 1)	
Sublabialia	10–12	9–12	9–13
	(10.71 ± 0.59)	(10.72 ± 0.73)	(10.90 ± 0.72)
Gulars in a transverse row between the last two sublabialia	11–17	10–16	11–16
	(14.59 ± 1.54)	(13.78 ± 1.31)	(14.23 ± 1.34)
Gulars touching anterior inframaxillaries	Absent or 1–2	Absent or 1–2	1–2
	(1.24 ± 0.56)	(1.06 ± 0.35)	(1.59 ± 0.50)
Gulars between posterior inframaxillaries	Absent or 1–4	Absent or 1–4	2–5
	(2.53 ± 1.07)	(2.52 ± 0.83)	(2.45 ± 0.80)
Dorsalia and temporalia touching parietals	9–14	9–20	9–15
	(11.37 ± 1.67)	(13.11 ± 2.11)	(11.32 ± 1.49)
Preventrale	2–6	Absent or 1–3	Absent or 1–3
	(3.71 ± 1.38)	(1.41 ± 0.83)	(1.23 ± 0.61)
Ventralia	200–222	194–222	190*–211 (201.81 ± 5.29)
	(208.60 ± 5.95)	(206.57 ± 5.84)	(199.64 ± 11.42)
Dorsalia	211–230	NA	NA
	(218.00 ± 6.36)		
Dorsalia at forebody	23 (n = 2), 25 (n = 15)	21 (n = 2), 22 (n = 2), 23 (n = 5), 24 (n = 4), 25 (n = 12), 27 (n = 1)	23 (n = 1), 24 (n = 2), 25 (n = 19)
Dorsalia at midbody	21 (n = 2), 23 (n = 8), 25 (n = 10)	23 (n = 6), 24 (n = 4), 25 (n = 50), 27 (n = 1)	23 (n = 4), 24 (n = 2), 25 (n = 26)
Dorsalia at hind body	19 (n = 15), 21 (n = 2)	18 (n = 1), 19 (n = 19), 20 (n = 4), 21 (n = 1)	18 (n = 1), 19 (n = 21)
Subcaudalia	57–78	61–79	60–74
	(66.81 ± 5.33)	(70.84 ± 5.29)	(67.31 ± 4.08)
Anale	2	2	2

Measurements (in mm)	*Elaphe druzei* sp. nov	*E. sauromates*	*E. urartica*
	(n = 22)	(n = 63)	(n = 20)
Snout-vent length	325–1541	278–1250	260–970
	(1066.80 ± 376.22)	(778.58 ± 289.31)	(683.66 ± 216.49)
Tail length	52–294	54–323	50–245
	(189.81 ± 81.34)	(175.66 ± 74.97)	(158.23 ± 60.47)
Total length	377–1835	334–1503	310–1205
	(1228.29 ± 459.17)	(937.25 ± 351.87)	(841.88 ± 274.17)
Rostrum height	2.19–6.31	2.05–6.90	2.80–6.08
	(4.56 ± 1.17)	(4.76 ± 1.45)	(4.06 ± 0.75)
Rostrum width	3.43–9.48	3.10–10.24	4.00–8.02
	(7.14 ± 1.71)	(6.71 ± 1.94)	(6.04 ± 1.49)
Inter-nostril distance	4.51–10.79	2.40–10.54	4.30–8.88
	(8.72 ± 2.06)	(7.21 ± 2.20)	(6.23 ± 1.56)
Loreal length	2.14–4.63	NA	NA
	(3.29 ± 0.80)		
Eye diameter	3.51–7.49	3.40–6.54	3.35–6.62
	(5.55 ± 1.18)	(4.99 ± 1.01)	(4.42 ± 0.85)
Head length	15.00–44.00	14.30–38.70	16.85–31.49
	(30.09 ± 7.56)	(25.02 ± 6.44)	(23.86 ± 5.81)
Head width	7.53–24.00	10.90–22.80	16.64–21.53
	(15.71 ± 4.70)	(19.13 ± 3.91)	(19.62 ± 1.74)
Head height	7.15–21.83	NA	NA
	(14.67 ± 3.83)		
Supraoculare width	2.44–5.99	2.00–6.90	2.80–5.08
	(4.72 ± 1.109)	(4.31 ± 1.39)	(3.81 ± 0.73)
Frontale length	3.94–9.51	4.30–12.50	4.80–8.48
	(6.76 ± 1.45)	(7.98 ± 1.91)	(6.67 ± 1.31)
Frontale width	4.79–8.09	3.50–8.60	3.5–6.7
	(8.00 ± 1.51)	(5.91 ± 1.45)	(5.27 ± 1.15)
Anterior inframaxillaries length	5.32–14.24	4.50–14.06	6.70–9.70
	(10.32 ± 2.74)	(8.43 ± 2.70)	(7.85 ± 0.85)
Posterior inframaxillaries length	3.84–10.96	3.40–12.50	5.20–8.45
	(7.75 ± 2.14)	(7.41 ± 2.49)	(6.38 ± 1.15)

Data presented as minimum–maximum (average ± standard error); n = number of examined specimens.

*Referring to the paratype of *E*. *urartica* from Azerbaijan (AZ IZANAS 518) that was incorrectly stated as having 154 ventral scales in Jablonski et al.^25^ and the number was corrected by Bunyatova, in lett. (2022).

**Table 3:** Summary of the variation of meristic characters and measurements in males, females, and juveniles of *Elaphe druzei* sp. nov.

Meristic characters	Males	Females	Juveniles
	(n = 6)	(n = 10)	(n = 2 for most of characters; n = 4 for a few)
Preoculare	1–2	1–2	1; 2
	(1.20 ± 0.45)	(1.33 ± 0.50)	
Postoculare	2–3	2	2
	(2.20 ± 0.45)		
Temporale	2–3	2–3	2; 3
	(2.40 ± 0.55)	(2.33 ± 0.50)	
Posttemporale	2–4	2–4	4
	(3.20 ± 0.84)	(3.44 ± 0.73)	
Supralabialia	8	8–9	8
		(8.10 ± 0.32)	
Supralabialia contacting the eye	4th and 5th (n = 4)	4th and 5th (n = 4)	4th and 5th
		4th and 5th, and 5th and 6th (n = 1) 5th and 6th, and 4th and 5th (n = 1)	
Sublabialia	10–12	10–11	11; 12
	(10.80 ± 0.84)	(10.56 ± 0.53)	
Gulars in a transverse row between the last two sublabialia	11–16	12–17	14; 16
	(13.80 ± 1.92)	(14.78 ± 1.39)	
Gulars touching anterior inframaxillaries	1–2	1–2	1; 2
	(1.40 ± 0.55)	(1.22 ± 0.44)	
Gulars between posterior inframaxillaries	1–4	2–4	2; 3
	(2.60 ± 1.34)	(2.78 ± 0.67)	
Dorsalia and temporalia touching parietals	11–14	9–12	11; 14
	(12.60 ± 1.34)	(10.63 ± 1.19)	
Preventrale	3–6	2–6	2; 5
	(4.00 ± 1.41)	(3.86 ± 1.35)	
Ventralia	202–214	200–222	203–210
	(205.60 ± 3.51)	(211.20 ± 7.00)	(205.50 ± 3.11)
Dorsalia	211–222	212–230	211; 213
	(213.75 ± 3.10)	(220.86 ± 5.96)	
Dorsalia at forebody	25	23 (n = 1), 25 (n = 8)	23; 25
Dorsalia at midbody	23 (n = 3), 25 (n = 1)	21 (n = 2), 23 (n = 3), 25 (n = 5)	25
Dorsalia at hind body	19 (n = 3), 21 (n = 1)	19 (n = 8), 21 (n = 1)	19
Subcaudalia	66–78	57–70	65–72
	(69.80 ± 4.76)	(63.83 ± 5.27)	(69.25 ± 3.10)
Anale	2	2	2

Measurements (in mm)	Males	Females	Juveniles
	(n = 6)	(n = 10)	(n = 2 for most of characters; n = 4 for a few)
Snout-vent length	980–1541	736–1356	325–372
	(1236.67 ± 222.99)	(1134.80 ± 216.16)	(342.33 ± 25.81)
Tail length	146–294	67–260	52–79
	(255.67 ± 56.33)	(179.73 ± 59.69)	(67.33 ± 13.87)
Total length	1126–1835	877–1530	377–443
	(1492.33 ± 267.14)	(1314.53 ± 225.44)	(404.75 ± 28.69)
Rostrum height	4.32–5.76	3.34–5.50	2.19; 270
	(5.25 ± 0.65)	(4.52 ± 0.66)	
Rostrum width	6.95–9.48	5.81–8.12	3.43; 3.87
	(8.39 ± 0.74)	(7.28 ± 0.88)	
Inter-nostril distance	8.86–10.79	6.77–10.68	4.51; 4.84
	(9.72 ± 0.83)	(9.12 ± 1.31)	
Loreal length	2.95–4.39	2.62–4.63	2.14; 2.35
	(3.57 ± 0.71)	(3.48 ± 0.81)	
Eye diameter	5.11–7.49	4.50–6.90	3.51; 3.66
	(6.40 ± 0.98)	(5.54 ± 0.83)	
Head length	27.13–44.00	21.76–39.00	15.00; 15.43
	(33.54 ± 6.29)	(31.15 ± 5.42)	
Head width	13.7–23.00	10.38–24.00	7.53; 8.08
	(18.10 ± 3.37)	(16.14 ± 4.38)	
Head height	15.78–16.89	11.00–19.12	7.15; 7.26
	(16.29 ± 0.50)	(14.82 ± 2.41)	
Supraoculare width	4.69–5.92	3.62–5.14	2.44; 2.88
	(5.28 ± 0.54)	(4.81 ± 0.72)	
Frontale length	8.06–9.41	6.21–9.51	3.94; 4.08
	(8.92 ± 0.63)	(8.20 ± 1.29)	
Frontale width	6.51–8.09	5.81–8.94	4.79; 5.89
	(7.48 ± 0.69)	(7.15 ± 1.12)	
Anterior inframaxillaries length	9.81–14.24	7.77–12.93	5.32; 5.50
	(12.65 ± 1.22)	(10.58 ± 1.87)	
Posterior inframaxillaries length	7.24–10.46	5.69–10.96	3.84; 4.08
	(9.07 ± 1.26)	(8.18 ± 1.69)	

Data presented as minimum–maximum (average ± standard error); n = number of examined specimens. For two specimens (TAU-R 14382, and another from Halboun, Syria—no voucher) we were not able to determine sex.

**Table 4:** Diagnostic characters of all species of *Elaphe* (*Elaphe druzei* sp. nov.: own data; Jablonski et al.^25^ for *E. sauromates* and *E. urartica*; Qi et al.^4^ for all remaining species).

Species	Dorsalia	Ventralia	Subcaudalia	Sublabialia	Preoculare	Max SVL
***E. druzei*** sp. nov	25 (23–25)-23, 25 (21)-19 (19–21)	200–222	57–78	11 (10–12)	1 (1–2)	1541
*E. anomala*	23 (21–25)-23 (19–23)-19 (17–19)	203–225	45–77	9–11	2 (1–2)	1925
*E. bimaculata*	23 (23–25)-23 (21–25)-19 (21)	170–209	61–81	9–12	2 (1–2)	760
*E. cantoris*	19 (19–21)-19 (19–23)-17	226–239	78–87	9–10	2	1158
*E. carinata*	23 (21–25)-23 (21–25)-19 (17–19)	186–227	69–102	9–12	2 (1–2)	> 2000
*E. climacophora*	NA-23 (23–25)-NA	222–236	97–116	11	2	> 1500
*E. davidi*	25 (22–27)-23 (22–25)-19 (17–21)	155–183	53–72	11–13	2 (1–3)	1227
*E. dione*	25 (21–27)-25 (21–27)-19 (17–21)	168–206	51–84	9–11	2 (1–2)	893
*E. hodgsoni*	23 (21–25)-23 (21–25)-17	228–247	72–92	9–12	2 (1–2)	1190
*E. moellendorffi*	25 (23–27)-27 (25–27)-19 (19–21)	270–278	92–102	10–13	2	1602
*E. quadrivirgata*	NA-19-NA	195–215	70–96	11	2	> 1000
***E. quatuorlineata***	25–25 (23–27)-19	187–234	56–90	11	2 (2–3)	> 2000
***E. sauromates***	(21–27)-(23–25)-(18–21)	199–222	61–79	9–12	1–3	1250
*E. schrenckii*	23 (21–23)-23 (21–23)-19	208–224	57–75	8–11	2 (1–2)	1335
*E. taeniura*	25 (23–25)-23 (21–25)-19 (17–19)	223–261	73–121	9–13	2 (1–2)	> 2000
***E. urartica***	(23–25)-(23–25)-(18–19)	190–211	60–74	10–13	1–3	970
*E. xiphodonta*	21–21-17	202–204	67–68	9–10	2	785
*E. zoigeensis*	21–19 (19–21)-17	202–212	68–79	9	3	722

The most often observed condition of the character is followed by its variation in parentheses in dorsalia, sublabialia, and preoculare. Members of the *E*. *quatuorlineata* group are in bold.

Max SVL, Maximum snout-vent length (in mm); NA, Not available.

#### Systematic account

Our integrative results from the molecular phylogeny, dating, genetic distances, and species delimitation analyses, combined with evidence from morphology, historical biogeography, ecology, and distribution, confirmed the existence of the three clearly distinguished clades previously found^25,27,28^. These are called *Elaphe quatuorlineata* (Bonnaterre, 1790) (type material and locality unknown), *E*. *sauromates* (Pallas, 1814) (type locality Pre-Sivash area of the Crimean Peninsula, the Perekop Isthmus, and adjacent territories of the Lower Dnieper region, with unknown type material), and *E*. *urartica* Jablonski, Kukushkin, Avcı, Bunyatova, Ilgaz, Tuniyev, Jandzik, 2019 (Kısıklı Village, Süphan Mts., Turkey, ZDEU 26/2012). Additionally, we detected here a fourth, unnamed clade. This forth clade from the Southern Levant is completely allopatric and separated from the nearest *Elaphe* populations by an airline ~ 400 km gap. The clade represents a cryptic evolutionary species that is morphologically differentiated and currently reproductive isolated from the other three species in the group. Thus, in accordance with the definition of the genetic species concept^35^, the evolutionary species concept (populations with a long independent evolutionary history, representing a lineage of ancestral descendent populations), and traditional morphological taxonomy^36,37^, that all support its evolutionary independence^38^, we describe this clade as a new species.

#### *Elaphe druzei* sp. nov. Jablonski, Ribeiro-Júnior & Meiri

The proposed common name in English, Hebrew, and Arabic: Levant Rat Snake, .

The LSID for the species: urn:lsid:zoobank.org:act:5BD3681F-3921-482C-9506-4823585680BC.

#### Holotype

**Figure 6:**
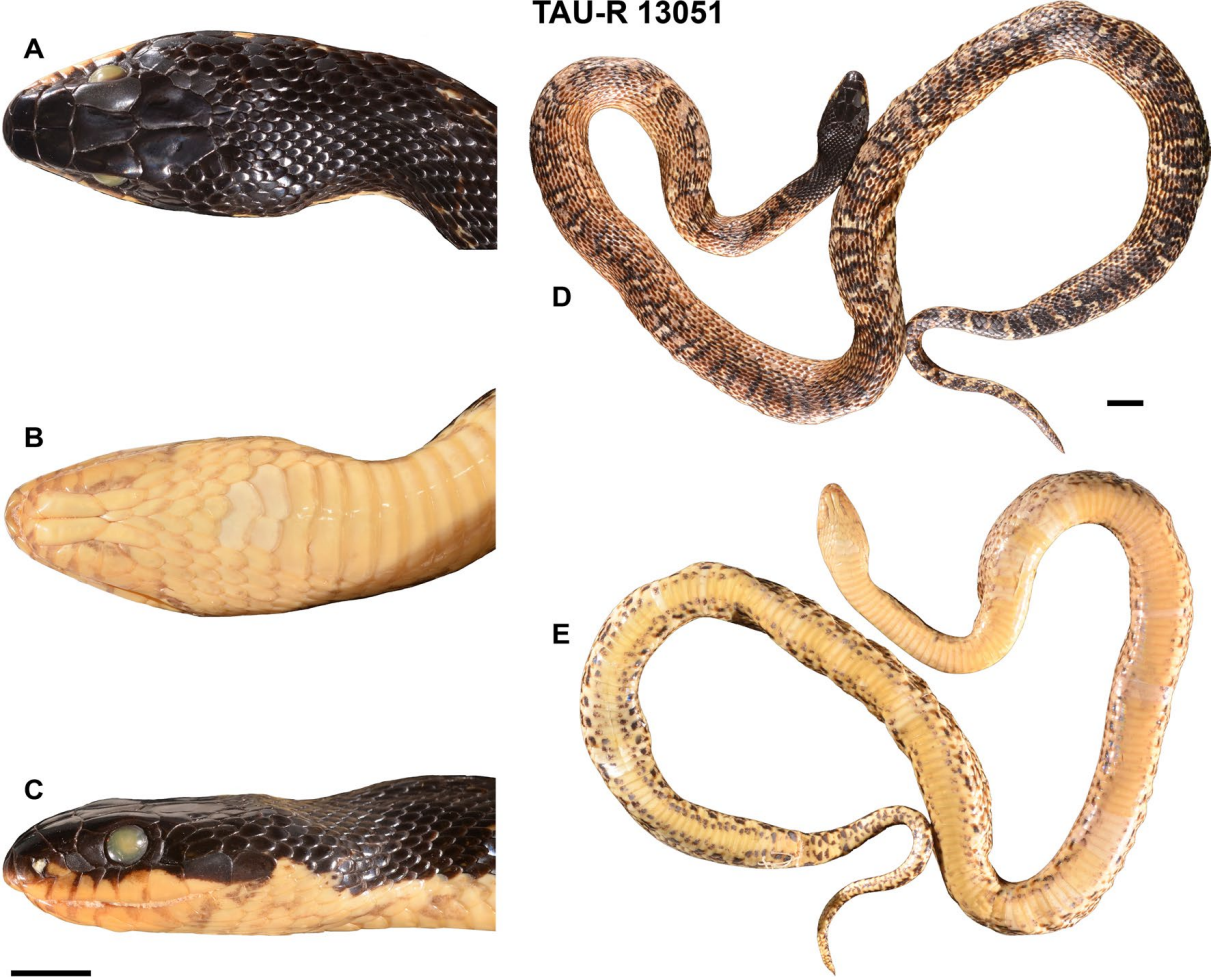
The holotype (TAU-R 13051) of *Elaphe druzei* sp. nov. (**A**) dorsal, (**B**) ventral and (**C**) lateral views on the head and body from the dorsal (**D**) and ventral parts (**E**). Bar = 1 cm (photographs by Marco Antônio Ribeiro-Júnior).

**Figure 7:**
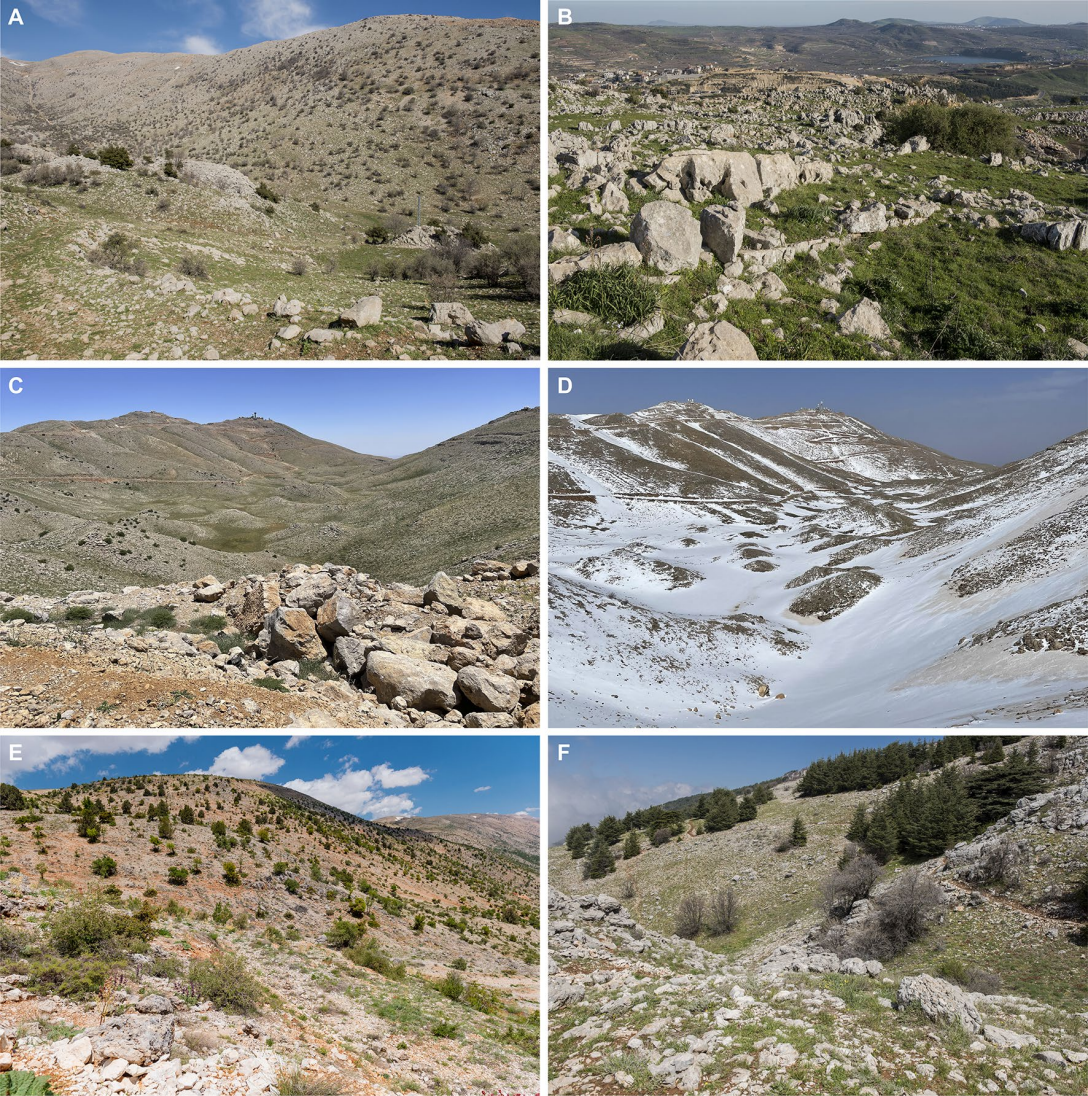
The spring (**A**–**C**) and winter (**D**) views on the habitat in Mount Hermon near Majdal Shams, Golan Heights, the type locality of *Elaphe druzei* sp. nov. and character of the habitat of the species in the Yammoune area (**E**) and Maasser Cedar Forest (**F**) both in central Lebanon. Photographs by Daniel Jablonski (**A**, **B**, **E**, **F**) and David David (**C**, **D**).

TAU-R 13051, adult female (Fig. 6); collected on 18 August 1983 at Majdal Shams, Hermon, Israeli-controlled Golan Heights (33.27° N, 35.77° E; 1100 m elevation; Fig. 7), collected by Yaakov Pessah.

#### Paratypes

TAU-R 19070 (DNA sample CUHC 9363; Table S2), adult male, dead body found on 16 May 2019 at Mount Hermon, Israeli-controlled Golan Heights (33.29°N, 35.77°E; altitude 1602 m) collected by Eran Levin (Fig. S5). TAU-R 19145, adult female, collected on 22 June 1973 at Mount Hermon, Israeli-controlled Golan Heights (33.30°N, 35.77°E; altitude 2100 m) collected by the Israel Defense Forces (Fig. S6). TAU-R 19438, adult female, collected in June 1971 at a few kilometres above Majdal Shams on the southern slope of Mount Hermon, Israeli-controlled Golan Heights (33.27°N, 35.76°E), collected by Yossi Levari (Fig. S7)^12^.

#### Diagnosis

*Elaphe druzei* sp. nov. is distinguished from all other species of *Elaphe* by the combination of the following characters (Table 4): (1) 200–222 ventralia, mean = 208.60; (2) 57–78 subcaudalia, mean 66.81; (3) 10–12 sublabialia, mean = 10.71; (4) mostly one preoculare (one, n = 11; two, n = 6); (5) mostly 25 (23–25) dorsalia at forebody; (6) 23 or 25 (rarely 21) dorsalia at midbody; (7) mostly 19 (rarely 21) dorsalia at hind body; (8) snout–vent length (maximum 1541 mm; mean = 1066.80 mm); (9) the dorsal surfaces of head, neck and anterior-most part of body homogeneous black in adults; (10) the lateral surface of the head, above supralabialia, homogeneously black in adult specimens; (11) the dorsal surface of the body yellowish with wide, oval black blotches filled by brown; (12) dorsal scales on body distinctly keeled and elongated; (13) dorsolateral scales on body slightly elongated, rather smooth; (14) the base of the hemipenis is ornamented by long pines, becoming drastically spinulated calyces on about ½ of the total organ length (on the contact area between body and base); (15) the apical area and the body of the hemipenis with several, conspicuous spinulated calyces.

#### Comparison with other *Elaphe*

*Elaphe druzei* sp. nov. differs from *E. cantoris*, *E. climacophora*, *E. hodgsoni*, *E. moellendorffi*, and *E. taeniura* by having 200–222 ventralia and 57–78 subcaudalia (vs. 226–239 and 78–87 in *E. cantoris*; 222–236 and 97–116 in *E. climacophora*; 270–278 and 92–102 in *E. moellendorffi*, respectively). It also differs from *E. davidi*, *E. hodgsoni*, and *E. taeniura* by having 200–222 ventralia (vs. 155–183 in *E. davidi*; 228–247 in *E. hodgsoni*; 223–261 in *E. taeniura*); from *E. quadrivirgata*, *E. xiphodonta*, and *E. zoigeensis* in having dorsalia counts as 25 (23–25)-23, 25 (21)-19 (19–21) (vs. 19 dorsalia on midbody in *E. quadrivirgata*; 21 and 17 dorsalia on anterior and posterior body respectively, in *E. xiphodonta*; and 21–19 (19–21)-17 in *E. zoigeensis*); it also differs from *E. zoigeensis* by having 10–12 sublabialia and one, rarely two, preoculare (vs. nine and three respectively). *Elaphe druzei* sp. nov. differs from *E. anomala*, *E. bimaculata*, *E. carinata*, *E. dione*, and *E. schrenckii* in that the dorsal surfaces of the head, neck, and anterior-most part of body are homogeneous black in adults, and the dorsal surface of the body yellowish with wide, oval black blotches filled by brown [vs. dorsal surface of body with wide and transverse black bands in *E. anomala* and *E. schrenckii*; head and body yellow, with transverse and inconspicuous black bands, in *E. carinata*; head and body light brown-greyish, with short and narrow brown stripes on dorsal surface of the body in *E. bimaculata*, and with short and narrow black stripes (or blotches) on dorsal surface of body in *E. dione*]. The maximum snout-vent length known for *E*. *druzei* sp. nov. is about twice the maximum for *E. bimaculata*, *E. xiphodonta*, and *E. zoigeensis* (Table 4).

*Elaphe druzei* sp. nov. differs from the other species of the *E. quatuorlineata* group (*E*. *quatuorlineata*, *E. sauromates*, *E. urartica*) by having distinctly keeled and elongated dorsal scales, and slightly elongated, rather smooth dorsolateral scales (Fig. 4A,B) versus oval and rather smooth dorsal scales and smooth, slightly elongated dorsolateral scales in *E*. *urartica* (Fig. 4C,D); feebly keeled and rather elongated and oval dorsal scales, and smooth, oval dorsolateral scales in *E*. *sauromates* (Fig. 4E,F); and diamond, feebly keeled dorsolateral scales in *E*. *quatuorlineata* (Fig. 4G,H). It also differs from *E*. *quatuorlineata* (Fig. 3) in having the dorsal surfaces of head, neck, and anterior-most part of body homogeneous black, and the dorsal surface of the body yellowish with wide, oval black blotches filled by brown, in adult specimens (vs. head and body brownish, with four longitudinal black stripes, two of them along the dorsolateral surface of body, and another two on the flanks in *E. quatuorlineata*). *Elaphe druzei* sp. nov. also differs from *E. sauromates* and *E. urartica* (see Jablonski et al.^25^) by having one preocular (65%, n = 17; rarely two, with the second one very small, between supralabialia, eye, and the large preocular, in a subpreocular position) [vs. two or three large preocular scales (rarely one); Fig. 3], and by having the lateral surface of the head, above the supralabialia, homogeneously black in adult specimens (vs. a yellow stripe from the upper loreal region, through upper eyes and medial temporal region, to the lateral surface of neck; Fig. 3). Clear differences are also conspicuous between juveniles of different species in pattern and coloration (Fig. 3).

Based on hemipenial morphology (Fig. 5), *E*. *druzei* sp. nov. differs from *E. sauromates* by having the base of the organ ornamented by long pines, becoming drastically spinulated calyces on about ½ of the total organ length (on the contact area between body and base) (vs. the spines on the base of the organ becoming gradually smaller, smoothly transforming in spinulated calyces), and from *E. quatuorlineata* by having the apical area and body with several evident spinulated calyces [vs. the body of the organ with spinulated calyces less pronounced, and the apical part has a smoother aspect (calyces are less evident and developed)].

#### Description of the holotype

*Elaphe druzei* sp. nov. TAU-R 13051, adult female (Fig. 6). Body cylindrical, snout-vent length 946 mm, tail length 176 mm. The head is long (26.55 mm) and wide (13.60 mm), clearly distinct from the neck. Head scales are smooth, and body scales keeled. Rostral slightly curved toward the top of the head, indistinctly wedged between the internasals. Rostrum height 4.43 mm and width 6.70 mm, in contact with the first supralabials, nasals and internasals. Nostrils located within the nasal scales, inter-nostril width is 8.91 mm. Loreal on either side of the head in contact with second and third supralabials. Two preoculars on each side of the head; lower one small, almost indistinct, bordering third and fourth supralabials dorsally, but not protruding between them. Two postoculars on each side of the head; the upper one about half the size of the lower one. Eyes circular with circular pupil of 5.12 mm diameter. Frontal distinct wider anteriorly; length 9.07, anterior width 5.87, posterior width 3.72 mm. Eight labials on each side of the head; fourth and fifth in direct contact with the eyes. Ten infralabials on each side of the head; first five in direct contact with the anterior chin shields. Two temporals on each side of the head; three post temporals on the left side, and four on the right. Eleven temporal and dorsal scales surrounding parietals, four of them on the posterior margin of the parietals. One gular scale in contact with the anterior pair of chin shields. A total of 25 dorsal scale rows at the level of one head length posterior to the head, 23 at the midbody, and 19 at one head length anterior to the cloaca level. Four longitudinal rows of chin shields, and two rows of paired pre-ventral scales. Ventral plates 210, paired preanal scales, and 61 paired subcaudals. Dorsal head, neck and anterior-most surface of body are homogeneous black. Upper lateral head, neck, and anterior-most surfaces of the body homogeneous black, and lower lateral surfaces yellowish. The dorsal surface of the body is yellowish with wide, oval black blotches filled with brown; on anterior and posterior surfaces of the body the blotches are paired. Flanks yellowish, with small rounded black blotches filled with brown; blotches are evident on anterior most and posterior most flank surfaces, and almost indistinct along the medial surface. Dorsal and lateral surfaces of the tail with similar botches, but darker and irregularly connected longitudinally. Ventral surfaces of the head, neck, body, and tail are yellowish, with irregular black spots on the ventrolateral surface.

#### Variation

**Figure 8:**
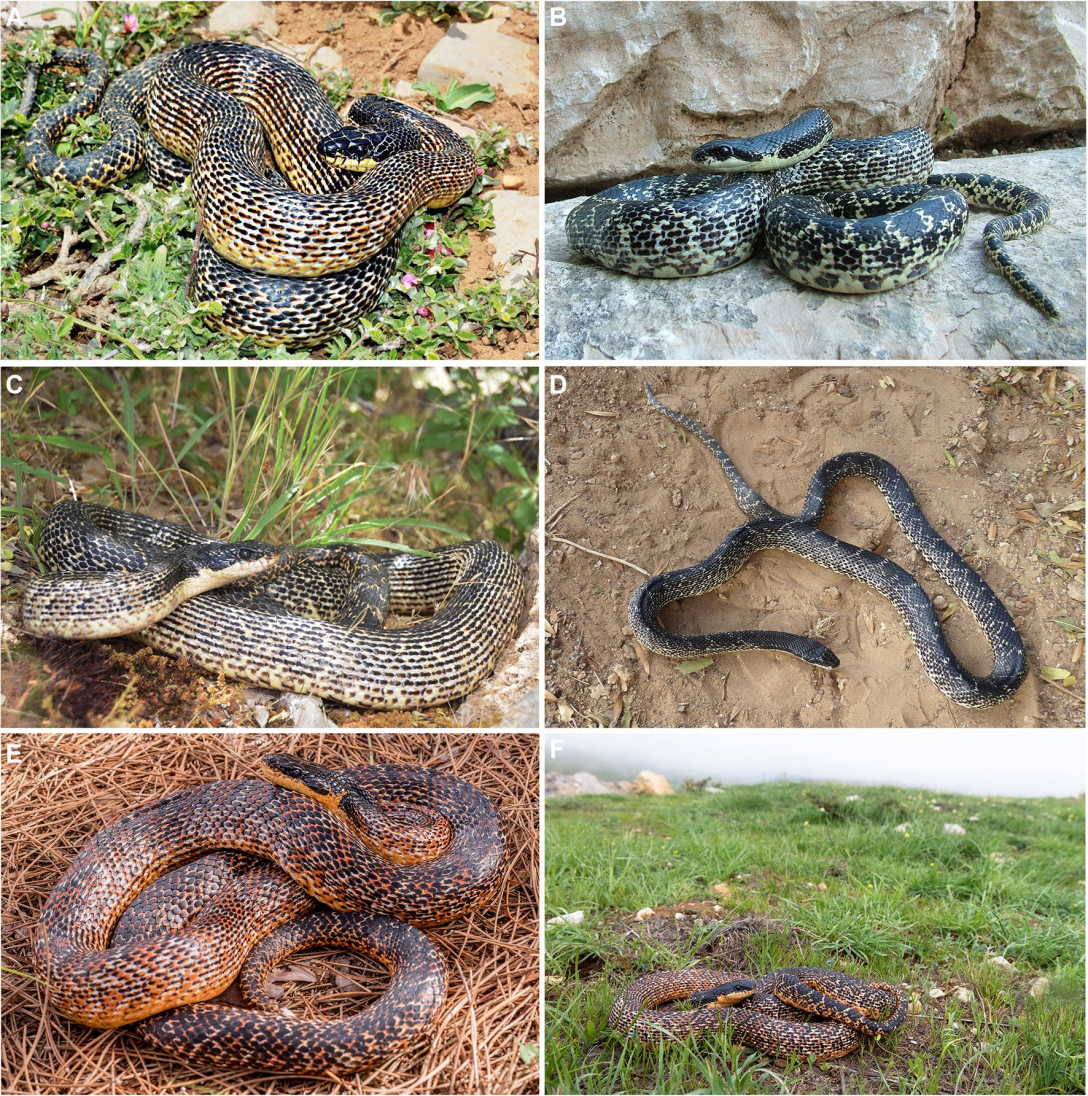
Colour and pattern variation in *Elaphe druzei* sp. nov. (**A**) adult individual from Mount Hermon (24 April 2015, photography by David David), (**B**) adult individual in captivity at the Zoological Garden in Naharyia originated from Mt. Hermon (photography by Aviad Bar), (**C**) adult individual from Mt. Hermon (28 May 2021, photography by David David), (**D**) adult male from Tel Aviv University Zoological garden originated from Mt. Hermon (21 November 2019, photography by Erez Maza), (**E**) adult female from Barouk, Kfar Slouan, Lebanon (April 2018) and (**F**) adult female from Mtein, Zaarour, Lebanon and its natural habitat (May 2022, photographs by Daniel Jablonski).

Table 3 presents a summary of the variation of meristic characters and measurements for *E*. *druzei* sp. nov. All male specimens from Lebanon have a second, small, subpreocular, while among six Israeli male specimens, only one specimen has it, and on only one side of the head (TAU-R 14131). One specimen (TAU-R 14131) has three postoculares; all others have two postoculares. The juvenile, TAU-R 11147, and the female CUHC 11712 have 23 dorsalia at the forebody, while all others have 25. One male, TAU-R 11463, and one female, CUHC 11712, have 21 dorsalia at the hind body, but all others have 19. Lebanese specimens have proportionally longer head than Israeli specimens (Israeli, n = 10, head length/snout–vent length = 0.021–0.029; vs. Lebanese, n = 4, head length/snout–vent length = 0.032–0.045). The adult female, NMW 23472 (Fig. S13) from Syria has a longer tail than all other adult female specimens (260 mm, vs. 67–230 mm for all others). Colour pattern: specimens TAU-R 13149, 14130, 14382, 17168, 19070 (paratype, Fig. S5), 19514 have the dorsal surface of the body and tail darker than the holotype (almost completely black), with the black blotches filled with black (compare Fig. 6 vs. Fig. S5). Other specimens from the TAU collection have a similar colour pattern to the holotype. Some investigated individuals (CUHC 6791, 11712, and available photos) from Lebanon, but not from Mt. Hermon, have a distinctly bronze or orange coloration, especially in the ventral and lateral parts of the body (Figs. 8E,F, S8, S9). The juvenile specimens TAU-R 11147 and 19144 (Fig. S12) have dorsal surfaces of the head and neck brown, with small cream patches along the dorsolateral surface of the anterior head; one large-rounded cream dot on the dorsal surface of the neck. The lateral surface of the head has a cream and oblique stripe, running from the anterior margin of parietals to the posteroventral surface of the neck (Fig. S12). On the dorsal surfaces of the body and tail and on flanks, the blotches are dark brown, inconspicuously bordered by black. Ventral surfaces of head, neck, body, and tail cream, with irregular brown spots on the ventrolateral surface (Fig. S12). For the variability in coloration of living individuals see Fig. 8.

Sexual dimorphism in *E*. *druzei* sp. nov. can be observed by longer snout–vent (SVL) and tail (TL) in males (SVL = 980–1541 mm, mean = 1236.67 mm; TL = 146–294 mm, mean = 255.67 mm) than in females (SVL = 736–1356 mm, mean = 1134.80 mm; TL = 67–260 mm, mean = 179.73 mm). Males also have larger heads (length, and height) than females (means = 33.54, and 16.29; vs. means = 31.15, and 14.82, respectively), and weigh more (632–958 g, mean 743 g, n = 4, vs. 321–895, mean 644 g, n = 3 in females). One zoo animal (TAU-R 14382, possibly a female) is even heavier (1394 g) but  may have been obese because of the captive conditions. Males have more dorsalia and temporalia touching parietals and subcaudalia (means = 12.60, and 69.80, respectively) than females (means = 10.63, and 63.83, respectively). We did not perform statistical tests due to the small sample size; these differences, therefore, remain to be tested quantitatively with more data obtained in the future.

#### Hemipenis

The hemipenis of *E*. *druzei* sp. nov. (TAU-R 13149) is “medium formed” (Hemipenis Proportion Index; HPI = 0.36), slightly bilobed, bulbous, and noncapitate (Fig. 5). The apical area presents several spinulated calyces (decreasing in size toward lobes), and between the lobes (medial part) the area is nude; a nude area is also found on the upper-medial asulcal side. *Sulcus spermaticus* is undivided and terminates laterally. The body is covered by spinulated calyces, gradually decreasing in size toward the apical part. The base is ornamented by 6–8 transverse rows of long pines, becoming drastically spinulated calyces on about ½ of the total organ length (on the contact area between body and base). Measurements: width 21.23; length 58.82. Hemipenis Proportion Index (maximal width of the hemipenis divided by total length): 0.36. A hemipenis with HPI > 0.5 is considered “stubby”; HPI between 0.5 and 0.25, “medium formed”; and HPI < 0.25, elongated^39^. In general aspects, the hemipenis of *E. druzei* sp. nov. is very similar in general aspects to those of *E. quatuorlineata* and *E. sauromates* (unfortunately no data are available for *E*. *urartica*), but a few differences can be recognized. The spines on the base of the organ become gradually smaller, smoothly transforming into spinulated calyces on the body of the organ in *E. sauromates*; the body of the organ in *E. quatuorlineata* has spinulated calyces less pronounced, and the apical part has a smoother aspect (calyces are less evident and developed).

#### Etymology

No name is available for the rat snake population (*Elaphe*) from the mountains of the Levant. We hence suggest a new name, *Elaphe druzei*, dedicated to the ethnoreligious group of people from the Levant—the Druze. The Druze, similarly, to the new species described here, are present primarily in the mountains of northern Israel, southwestern Syria, and Lebanon. The oldest and most densely populated Druze communities are in Mount Lebanon which correspond to the main distribution range of the new species according to our data. Moreover, the first, verified record of the *E. quatuorlineata *group from the Levant^12^ is from the vicinity of a Druze town, Majdal Shams, that is also the type locality of *E*. *druzei* sp. nov. Interestingly, one of the biogeographic hypotheses on the origin of Druzes says that they might have originated in the Zagros Mountains and the surroundings of Lake Van from where they later migrated south to the mountains of Lebanon and Anti-Lebanon^33^. This represents certain parallelisms with the uncovered phylogenetic relationship between the new species and the sister *E*. *urartica* that has its centre of distribution in eastern Anatolia, with the type locality near to Lake Van^25^. The two species of *Elaphe* most probably diverged somewhere in Anatolia or the Middle East with subsequent independent evolution in eastern Anatolia and the Levant (see Discussion). Druze practice Abrahamic religion with secretive theology, however, they have taken their philosophy from different doctrines and religions including ancient Greek philosophers, Isma'ilism, Judaism, Christianity, Zoroastrianism, Buddhism, Hinduism, Persians, and others which emphasizes the role of the mind and truthfulness^40^. Moreover, Druze believe in seven prophets including Moses that is one of the most important prophets of all Abrahamic religions that live together in the Levant, and they have a reputation for being peaceful, cooperative, hospitable, and highly educated people. The Druze are also widely respected for their contributions to society, particularly in the fields of science and medicine. Considering the geographic origin of the new species (the western Middle East), and the type locality (the Hermon area that is shared among three countries), we consider it symbolic that the Druze represent a human group that is inspired by elements joining different origins and cultures. This is very topical in the still divided Middle East. Such a view can provide a space and inspiration for collaboration on conservation and research relating to the unique biota of the Levant, including this newly described, unique, rare, mountain, and endemic snake.

#### Distribution

During the late twentieth century, the common opinion was that the population of *Elaphe* from the Levant is conspecific with *E*. *quatuorlineata* (despite having completely different pattern and coloration) and later with *E*. *sauromates*^12,22,29,30^. With the description of a new species from eastern Anatolia and the Caucasus (*E*. *urartica*), Jablonski et al.^25^ suggested that the Levantine population may belong to this newly described species. On the other hand, they also opined that the Levantine population may represent a yet undiscovered taxon given the commonly observed uniqueness of reptiles, including snakes, occurring in Mediterranean regions south of the Nur Mountains and the Amik Basin in southern Turkey (e.g., *Xerotyphlops syriacus*, *Daboia palaestinae*, *Micrelaps muelleri*^41^, and the lizards *Phoenicolacerta laevis*^42^ and *Chalcides guentheri*). Some squamates (e.g., *Mediodactylus amictopholis, Montivipera bornmuelleri*) directly represent endemics of Lebanon and Anti-Lebanon Mountains with the southern edge of their distribution at or around Mount Hermon^30^.

*Elaphe druzei* sp. nov. is currently known to be distributed in Israel, Lebanon, and Syria (Table S1). It is limited to hilly and mountain areas in and around the southern Anti-Lebanon Mountains (mostly Mt. Hermon) and the southern and central Lebanon Mountains (Fig. 1A, B)^15,18,19,21,30,43^. The species distribution modelling (Fig. 1B) showed the most suitable habitats are in the foothills of the Lebanon Mountains (not at the highest elevations) and in mid elevations of the southern Anti-Lebanon Mountains (Hermon). The model also showed Mount Meron (ca 1200 m a.s.l.) in the upper Galilee, Israel, as a possible habitat. This area is well surveyed, but the species has never been recorded there. On the other hand, most of areas in central and northern Anti-Lebanon Mts. were assessed as unsuitable (but see^21^), probably due to their semi-desert environment. *Elaphe druzei* sp. nov**.**, according to our knowledge, is allopatric, and completely isolated from other species of the *E*. *quatuorlineata* group (see Fig. 1A, B and compare it with^25^). The overall expected area of distribution is ~ 3000 km^2^, according to our SDM, which means that this species has one of the smallest distribution ranges in the whole genus *Elaphe* (http://www.gardinitiative.org/^2^). Only *E*. *xiphodonta* and *E. zoigeensis* may have smaller ranges. We cannot exclude its presence in the little-studied Syrian Coastal Mountains and in southern Turkey (Nur or Amanos Mountains; see^12^ and distribution pattern of other Levant endemic taxa or clades^31,44–48^) but there are no reported sightings from these areas. The possible distribution of the species in Jordan (e.g., in the Yarmouk^49^) also needs further investigation. However, the reptiles that have their southern distribution in Mt. Hermon and the northern Golan Heights, including *Phoenicolacerta kulzeri* (also present on mountain peaks further south in Jordan), *M. amictopholis*, and *M. bornmuelleri*, are not, as far as is currently known, found in northern Lebanon, coastal Syria, or Turkey. *Eirenis lenvantinus* may be an exception, though it is also found west of the Hermon in northernmost Israel. Reptiles that do inhabit these regions, such as *Rhynchocalamus melanocephalus, M. muelleri, Eirenis rothii*, *E. lineomaculatus*, *E. decemlineatus*, *C. guentherii*, and *Phoenicolacerta laevis* seldom reach as high elevations on Mt. Hermon as *E. druzei *sp. nov., and all range well into central Israel^2,30^. A chronological overview of obtained distribution records of *E. druzei* sp. nov. in the Levant is presented in Table S1.

#### Ecology and habitat

Due to the overall rarity of *E*. *druzei* sp. nov. and its secretive lifestyle, very scattered information is available on this new species. This is exacerbated by the hardly accessible habitat it inhabits. Furthermore, the species range lies in a politically highly unstable region that is not easy to investigate. Although it is a large and morphologically distinctive species, in its natural environment it is well hidden^12^. It is a diurnal snake, found in vegetation-rich landscapes, observed on the ground, shrubs, and climbing in trees and often near water bodies (own data). It is strictly confined to mountain and submountain habitats (Fig. 7) according to literature and our own data (see Table S1). According to the results of our species distribution modelling, the variables that mostly account for the species presence are the mean temperature of warmest quarter (Bio10), precipitation of the wettest month, and of the coldest quarter (Bio 13 and 19, respectively), and elevation. The new species is distributed from 900-950 m a. s. l. (Al-Quneitra, camp Fauar, Syria, 1976;^15,50^) to 2200 m (Halboun, 30 km NW Damascus, 22 May 2020^21^). In Lebanon, it is known from the western slopes of Hermon and from cedar forests and meadows of the Lebanon Mountains in the elevation between 1400 and 1850 m a. s. l. (In den Bosch et al.^18^, Hraoui-Bloquet et al. ^20^ own data). In the Israeli part of Hermon and the Golan Heights it has been recorded from 1270 to 2100 m a. s. l. The habitat in Syrian Al-Quneitra is similar to that of the Hermon region as a whole^15,17^. Recent and currently northernmost record of the species from Syrian Anti-Lebanon Mts.^21^ describe that the species habitat is characterized by rocky hills of eroded soil with a very sparse typical Sub-Alpine steppe vegetation of short grass (*Bromus* sp., *Geranium* sp.), shrubs (*Acantholimon ulicinum*, *Astragalus hermoneus*, *Cerinthe minor*, and *Marrubium libanoticum*), and juniper (*Juniperus excelsa*). According to available data, the species is active from April [specimen AR-0881 (AUB 1) from Kfar Selwane, Lebanon; Fig. S10] to early October (TAU-R 14130, Hermon, Israeli-controlled Golan Heights) with most observations in June (Table S1). Sympatric reptile species inhabiting the range of *E*. *druzei* sp. nov. include *Testudo graeca*, *Mediodactylus amictopholis*, *Ptyodactylus puiseuxi*, *Laudakia vulgaris*, *Lacerta media*, *Ophisops elegans*, *Parvilacerta fraasi*, *Phoenicolacerta kulzeri*, *P*. *laevis*, *Ablepharus rueppellii*, *A*. *budaki*, *Chalcides guentheri, Eumeces schneiderii, Heremites vittatus*, *Dolichophis jugularis*, *Eirenis levantinus, E. rothii, Hemorrhois nummifer, Platyceps collaris*, *Telescopus fallax, Zamenis hohenackeri*, *Malpolon insignitus, Daboia palaestinae,* and *Montivipera bornmuelleri* (^18,20,21,30^; the Steinhardt Museum of Natural History collections, and our own observations). Interestingly, the unusual coloration pattern of *E*. *druzei* sp. nov. (orange-yellow shade zigzag) observed in individuals found from the Lebanon Mts. (Fig. S14A) resembles a pattern of sympatric *D*. *palestinae* (Fig. S14B). It may thus represent the mimicry imitative venomous snakes of the family Viperidae that are known in the genus *Elaphe*^4^. In Tel Aviv University’s Garden for Zoological Research, snakes kept in terraria lived nearly 14 years, fed on small vertebrates, and females laid clutches comprising 6–16 eggs^30^.

#### Conservation

The borders of Israel, Lebanon, and Syria (i.e., the Golan Heights), are under dispute. In the presented map (Fig. 1A,B) we included the territory of the Golan Heights, where the type locality of *E*. *druzei* sp. nov. lies (Fig. 7), as the region controlled by Israel. This does not signify any political intention on behalf of the authors and is not aimed to suggest one. That said, Israeli occupation of the Golan Heights, and its recognition of them as Israeli territory, makes Israel responsible for the conservation of the species in the Hermon area. As mentioned by Bar et al.^30^, this species is rare in the Golan and is likely threatened by increased land-use changes for tourism (e.g., the only ski resort in Israel is in Hermon), habitat degradation by military activities, overpopulation, accelerated development, traffic density, collecting, and, of course, like all Hermon endemics, by global warming. The abundance of the new species in Lebanon and Syria is unknown but, according to our data, it seems that the species is similarly rare in the whole known range and under threat (see the urbanization and destruction of natural habitats in Lebanon^51^). The political instability in the region is also not helping species conservation. Its relative rarity, isolation, and the hardly accessible (often army or militant-controlled) mountain habitats it inhabits are also reasons why the population in the Southern Levant has been discovered only 50 years ago^12^ despite previous zoological investigations^52^. However, sub-fossil data^13,14^ suggest the possible presence of *Elaphe* (that should most probably be the here-described new species) in northern Israel (the Hula Valley and Carmel) at least from the Pleistocene and into the Natufian period (~ 12,000 years ago).

Based on the size (~ 3000 km^2^) and the character of its range (that is probably fragmented; Fig. 1B), and overall species rarity, we suggest that this large snake could be considered Endangered globally based on Criteria B1a,b ii, iii and B2a,b ii, iii (EOO < 5000 km^2^, AOO < 500 km^2^, severely fragmented populations, estimated continuous declines in the area of occupancy, and decline in the quality of its habitat—based on the abovementioned threats). It is one of the rarest snake species in the western Palearctic and we infer that its population is declining. This fact should lead to strict species protection that will include awareness of local people from all covered countries. In Israel this species is protected by law, and virtually its entire distribution in Israeli-controlled territory is in a nature reserve, albeit one with intensive military, tourist, and cattle grazing activities. But it is not protected in Lebanon and Syria. The distribution of this species in three countries, two of which are officially in a state of war with the third, and suffering from a long civil war or political instability, make the much-needed mutual international collaboration unlikely at the short term, but highly desirable (see EcoPeace Middle East, http://ecopeaceme.org^53^) for the protection of high elevation habitats and local endemism, especially in the Hermon area. This species is non-venomous, non-aggressive, and harmless. Members of the *E*. *quatuorlineata* group are very popular as pets, and have been since ancient times^1,8^ where they were used for different religious and cultural purpouses (Fig. S15). However, snakes are often killed indiscriminately in the Levant, which was probably also the case with several specimens of *E*. *druzei* sp. nov. (TAU-R 19070, TAU-R 19438, TAU-R 19514) stored in the Steinhardt Museum of Natural History in Tel Aviv.

## Discussion

Based on our integrative approach, combining genetic and morphological data, biogeography, and ecology, we investigated the enigmatic population of the large member of genus *Elaphe* in the Levant. We showed that the Southern Levant region is a fourth centre of the evolution of the *E*. *quatuorlineata* group resulting in the origin of the unique clade, described here as *E*. *druzei* sp. nov.

### Evolution in the *Elaphe quatuorlineata* group

A new clade in the well-known genus *Elaphe* from the Western Palearctic helps us understand the past biogeographic dynamics of these popular and conspicuous snakes. Our divergence time estimations showed that the *E*. *quatuorlineata* group started diverging ca. 5 Mya according to full mitogenome sequences and 5.6 Mya according to Cyt *b* dataset from other congeners. This is in accordance with the previous estimates for the group^27,28^ although our hypothesis of initial and subsequent divergence is younger than in published studies. On the other hand, both these published estimations used only one marker and two (*E*. *quatuorlineata*, *E*. *sauromates*) of the four currently recognized species of this group.

The well-supported phylogenetic trees we reconstructed (Figs. 1, 2) clearly separate clades that are also biogeographically well defined. Although genetic distances inside this group (especially for the *16S* marker) are low compared to other *Elaphe* species^4,54^, and nuclear markers indicate possible incomplete lineage sorting (Fig. 1D), they still support the evolutionary independence of all four recognized clades. Furthermore, they are comparable to, or higher than, differences within recognized taxa from the *Vipera ursinii-renardi* complex^55^. The existence of four species is strongly supported by molecular dating, molecular PCAs, morphology, and biogeography (Fig. 2). We infer a main initial Miocene radiation of two clades: the Western (“European”, later forming *E*. *quatuorlineata*/*E*. *sauromates*) and an Eastern (“Middle Eastern”, *E*. *urartica*/*E*. *druzei* sp. nov.). The ancestral populations were probably separated by the Parathethys strait (Fig. 2C) during the Messinian salinity crisis. The western and southern Balkans (*E*. *quatuorlineata*) quickly separated (4.6 Mya in mitogenome/5.13 Mya in Cyt *b*) from the western Anatolia or Eastern Balkans populations (becoming *E*. *sauromates*). The Eastern Anatolia population (that become *E*. *urartica*) and the Levant population (that evolved into *E*. *druzei* sp. nov.) diverged later (3.4/3.9 Mya; Fig. 2D). We hypothesize that the area occupied by the ancestor of *E*. *urartica* and *E*. *druzei* sp. nov. has likely been larger in the past (see below). Separation between *E*. *urartica* and *E*. *druzei* sp. nov. may have occured in southern Anatolia, perhaps around the area of Nur Mountains acting as a biogeographic break^42,46–48,56^. Kornilios et al.^27^ discussed even older event that caused the split between *E*. *quatuorlinata* and *E*. *sauromates* than we present here, i.e., the braking-up of the southern Aegean landmass and population vicariant speciation.

The newly detected clade has the smallest range of all species of the *E*. *quatuorlineata* group without any current geographic contact with other *Elaphe* taxa. Having been isolated for a long time far from its sister species, *Elaphe druzei* sp. nov. likely form a reproducibly isolated species, the operational criterion for the delimitation of species according to the biological species concept^38^. According to the GARD database (http://www.gardinitiative.org/^2,57^) and the literature, the expected range for *E*. *quatuorlineata* is over 300,000 km^2^, for *E*. *sauromates* 1,680,000 km^2^, and *E*. *urartica* about 785,000 km^2^, while we estimated a range of only about 3000 km^2^ for *E*. *druzei* sp. nov. Comparing these sizes of the geographic range and known genetic diversity for all species^25,27,28^, we can raise several hypotheses regarding the Plio-Pleistocene phylogeography of the group. We divide these snakes into two groups according to genetic and morphological data, i.e., *E*. *quatuorlineata* and other taxa of the group. The genetic diversity of *E*. *quatuorlineata* is high—especially in the Aegean area. The species comprises several island endemic lineages probably originating in the late Pliocene and displaying high phenotype variability^58,59^. Other lineages of the species that diverged during the Pleistocene, are distributed in the European mainland and in Corfu Island, Greece^28^ (Fig. 2B). On the other hand, the three other species in the group show very low (*E*. *sauromates*) or almost no intraspecific genetic variability (*E*. *urartica*^25^, *E*. *druzei* sp. nov.; this study) and are morphologically similar. Such less genetic variability is surprising for the geographically well DNA sampled *E*. *sauromates* (but see nuclear genes) that has the largest distribution range of the group characteristic by the high topographic heterogeneity^57^. In view of the low genetic variability in three species considered in the past to be *E*. *sauromates* we hypothesize that their current ranges reflect relatively rapid dispersion events during the Late Pleistocene or even the Early Holocene (see also another large-sized reptile of the region, *Pseudopus apodus*^31^). Fossil records (as shown in Fig. 2E) of snake resembling the *E*. *quatuorlineata* group found in the Hula Valley 13 (at approximately 70 meters above sea level) outside of the current range of the new species suggest that the extinction of peripheral populations in the past may have led to a loss of genetic variability. Thus, further sampling, and more fossil evidence, are needed from areas that were poorly or not studied to support or reject this hypothesis. Morphological variation is high in all four species, with colour patterns varying onthogenetically and geographically, probably reflecting different evolutionary history and local environmental conditions (Jablonski et al.^25^; Tables 2, 4, Figs. 3, 4, 8).

### Mountain endemism in the Levant

The Levant is well known as a major source of reptile endemism in the Western Palearctic^60,61^ at the subspecies (e.g., *P*. *apodus levantinus*), species (e.g., *Daboia palaestinae*), and genus levels (e.g., *Phoenicolacerta* spp.^30,31^). This could be related to rapid past environment changes which supported speciation and strong selection from Oligocene up to Pliocene^42,62–64^. The current high level of endemism is in the north–south direction between Nur Mountains and the Negev desert. Although a comprehensive biogeographic review of the endemic herpetofauna of the Levant is missing, this endemism could be generally further divided between taxa (i) that currently occur in the lowlands and/or middle elevations, and have wide distribution through the Levant (e.g. *Ptyodactylus puiseuxi*, *Phoenicolacerta laevis*, *Ablepharus rueppellii*, *Chalcides guentheri*, *P*. *apodus levantinus*, *Xerotyphlops syriacus*, *Rhynchocalamus melanocephalus*, *D*. *palaestinae*, *Micrelaps mulleri*) and (ii) to species that are endemic to higher elevation habitats of local mountains (*Parvilacerta fraasii*, *Phoenicolacerta kulzeri*, *Mediodactylus amictopholis*, and *Montivipera bornmuelleri*). These mountain endemic species are characterized by small, often fragmented distributions, located in the Anti-Lebanon (including Mt. Hermon), and Lebanon Mountains (Fig. 1B) (also the Jordanian highlands in the case of *P*. *kulzeri*). *Elaphe druzei* sp. nov., as shown by our data, belongs to the second group, representing a genetically divergent, endemic, homogenous, and evolutionary independent clade.

Tamar et al.^42^ investigated the phylogeography of the genus *Phoenicolacerta* and discussed the mid-late Miocene radiation of two well-supported and major clades with the subsequent diversification of the monophyletic *P*. *kulzeri* clade back to the Pliocene/Pleistocene. However, the inner genetic diversity, including both mitochondrial and nuclear markers, in this clade is not deep. This resembles the situation detected here in *E*. *druzei* sp. nov. although the split of the new snake clade is younger (Early Pliocene). The small, fragmented area of the current distribution, and low intra-clade genetic diversity of *P*. *kulzeri* and *E. druzei* sp. nov., suggest that climatic fluctuations might have reduced their genetic variability (i.e., local population extinctions)^56^. In such case, local populations of these mountain species could have been more widespread during the cold periods of the Pleistocene and were restricted to mountains during the warmer interglacial periods (cold-tolerant biota in sky-islands interglacial refugia^65^). Today populations are separated from others by valleys with unsuitable habitat or climatic conditions. This pattern was also observed in other reptiles in the mountains of the Mediterranean area^66,67^. Surprisingly, results of mitochondrial phylogenetic relationships of a mountain endemic viper *M*. *bornmuelleri* (the Pliocene origin), showed deeper structuring of analysed populations (although the sampling was very limited) between the Hermon and Syrian populations^68^. We only genetically analyzed two populations: Hermon and the Lebanon Mountains ones. They are genetically almost identical to each other for all investigated markers. We cannot exclude the possibility that wider sampling, and the inclusion of other localities, may reveal deeper genetic structuring in *E*. *druzei* sp. nov. but this is rather unlikely.

## Material and methods

Due to the overall rarity of snakes of the studied population, we were only able to use tissue samples from four specimens of Southern Levant *Elaphe*: two from Hermon (Anti-Lebanon Mountains; voucher number TAU-R 14131, associated with DNA tissue sample CUHC 6719, and TAU-R 19070 = CUHC 9363) and two from the Lebanon Mountains, Lebanon (CUHC 6791 and CUHC 11712, both living individuals; Table S1). These were used for all genetic analysis together with other sequences we generated and sequences available in GenBank (Table S2). We also newly sequenced samples of the holotype of *E*. *urartica* (ZDEU 26/2012 = CUHC 1124), and one representative of the Levant population (CUHC 6719), to obtain a full mitogenome sequence of species in the *E*. *quatuorlineata* group. We had the appropriate permissions to use the snake tissue voucher specimen from different museums. Genetic data from the Southern Levant populations were compared with sequences representing major clades of the group obtained from^25,26,28,34^.

We recorded morphological data from 22 specimens of the Southern Levant population deposited at the Steinhardt Museum of Natural History, Tel Aviv University (TAU-R 11147, 13051, 14130, 14131, 13149, 11463, 14382, 17168, 19070, 19144–45, 19514, 19438), American University of Beirut [AR-0881 (AUB 1), AUB 2; Figs. S10, S11], Natural History Museum, Vienna (NMW 23472; Fig. S13), available data of the Comenius University Herpetological Collection managed by the first author (CUHC 6791 and CUHC 11712, Figs. S8, S9) and literature data (^15,18,21,43^; Tables 2, 3, S1). No snakes were sacrificed for the present study. Differences in sample size among the tables stem from different numbers of specimens from which we could collect the listed characters in each table. Species comparisons were based on diagnostic characters for all species of this genus^4^.

Data on distribution and occurrence of the studied Levant population were collected from museum voucher specimens, literature, databases, citizen science portals (iNaturalist.org, gbif.org), and our own observations and sources (Table S1). All records were verified personally or using photographs.

### Genetics

#### Tissue sampling and laboratory procedures

As a source of DNA, we used blood (taken from the caudal vein), or saliva from live specimens, or liver, or muscle biopsies from ethanol-preserved museum specimens or road-killed snakes. We newly produced sequences of four mitochondrial and four nuclear markers of the currently recognized species of the *E*. *quatuorlineata* group as well as two full mitogenomes for *E*. *urartica* and the Levant *Elaphe*.

Total genomic DNA was extracted from the tissue samples using the E.Z.N.A.® Tissue DNA Kit (Omega Bio-tek, Inc., USA) and NucleoSpin Tissue Kit (Macherey-Nagel, Düren, Germany), following the manufacturer’s instructions. For molecular/genetic analyses, we newly generated and/or combine sequences of four mitochondrial genes, particularly *16S* rRNA (*16S*), cytochrome c oxidase subunit 1 (*COI*), the mitochondrial protein-coding segment of NADH dehydrogenase subunit 4 (*ND4*) (including the flanking tRNAs Serine, Histidine, and part of Leucine), cytochrome *b* (Cyt *b*) and four nuclear genes, i. e. the melanocortin 1 receptor (*MC1R*), the neurotrophin-3 (*NT3*), the nuclear protein-coding genes for the prolactin receptor (*PRLR*) and the recombination activation gene 1 (*RAG1*) following primers and conditions of PCR presented in^25,27,48^. We also obtained new sequences of *NT3* for *E*. *quatuorlineata* and *NT3*, *MC1R* and *RAG1* for the holotype of *E*. *urartica* that were not available previously^25^. The sequences obtained by Sanger sequencing were supplemented by marker fragments obtained from mitogenomes (see below), or those from previous studies on the group^25–28,34^. The *16S* and Cyt *b* fragments were also sequenced using primers from ^69,70^. The oocyte maturation factor Mos (*C*-*mos*) gene has not been newly sequenced but used for molecular phylogenetic analyses based on available data (see below). Molecular laboratory work was processed at the Department of Zoology, Comenius University in Bratislava, Slovakia, and PCR products were outsourced to Macrogen Europe (Amsterdam, The Netherlands). Details on the complete dataset of new and published sequences and their GenBank accession numbers are presented in Table S2.

To obtain complete mitogenome sequences for all taxa of the *E*. *quatuorlineata* group, we isolated total genomic DNA of samples CUHC 1124 (*E*. *urartica*) and CUHC 6719 (the Levant *Elaphe* population) using a commercial DNA Extraction kit (Qiagen DNeasyVR Blood and Tissue Kit, Venlo, Netherlands), according to the manufacturer’s instructions. DNA library preparation (0.5 ng total DNA) was carried out according to the Nextera XT DNA Library Prep Kit (Illumina, San Diego, CA) workflow. Sequencing was performed using Illumina MiSeq platform with MiSeq Sequencing kit version 3 (600 cycles, paired-ends reads; Illumina, San Diego, CA). For data analysis with CLC Genomics Workbench 9.5.2 (https://www.qiagenbioinformatics.com), 131 million (Levant population) and 723 thousand (*E*. *urartica*) of paired-end sequencing reads were first trimmed using quality filter 0.01. De novo assembly into contigs using strict parameters (Length fraction 0.8, Similarity fraction 0.9) followed by mapping to the mitochondrial genome of *E. dione* (KM065513 and see Simonov et al.^71^) enabled the identification of mitochondrial DNA represented by three (CUHC 6719) and ten (CUHC 1124) sequences that were assembled into a consensus sequence. The sample CUHC 1124 was assembled also by Geneious assembler and Spades and consensus sequences were aligned and merged. The consensus sequence of 6719 was aligned to the final 1124. All obtained consensus sequences were manually curated and gaps filled mapping the reads (Length fraction 0.3, Similarity fraction 0.8) to the obtained consensus sequence. The newly obtained mitogenome sequences were compared with available mitogenomes of snakes from the family Colubridae.

#### Phylogenetic analysis of mtDNA and nDNA markers

We checked the final sequences visually and aligned them using BioEdit 7.0.5.2.^72^. We performed a BLAST search in GenBank to confirm that the targeted loci were amplified. The translation of protein-coding sequences into amino acids was checked using DnaSP 6.00^73^ and the absence of stop codons was confirmed. Then we combined new sequences with GenBank data (Table S2). The final concatenated alignment used for subsequent phylogenetic tree analyses contained 23 sequence chains formed by nine markers. Seven sequence chains represent the *E*. *quatuorlineata* group (four representing Levant population) and 16 represent outgroup taxa. They have the following lengths in the concatenated alignment: 1–1353 bp (*16S*), 1354–2225 (*COI*), 2226–3106 (*ND4*), 3107–4225 (Cyt *b*), 4226–5241 (*RAG1*), 5242–5891 (*MC1R*), 5892–6458 (*C*-*mos*), 6459–7027 (*NT3*), and 7028–7578 (*PRLR*) (Table S3). With this dataset, we constructed Bayesian Inference (BI; MrBayes 3.2.6^74^) and Maximum Likelihood (ML; RAxML 8.0.0^75^) phylogenetic trees. The best-fit model of sequence evolution was selected using PartitionFinder 2^76^ with the following parameters: dataset divided into 25 subsets based on four user schemes (unpartitioned, gene-part, 3rd-pos-extra, codon-part). Final models for BI and ML analyses based on defined subsets are presented in Table S3. The BI analysis was set as follows: two separate runs, with four chains for each run, 10 million generations with trees sampled every 100th generation. The first 20% of trees were discarded as the burn–in after inspection for stationarity of log–likelihood scores of sampled trees in Tracer 1.7.1 (Rambaut et al.^77^; all parameters had effective sample sizes [ESSs] > 200). A majority-rule consensus tree was drawn from the post-burn-in samples and posterior probabilities were calculated as the frequency of samples recovering any clade. Nodes with posterior probability values > 0.95 were considered strongly supported. The ML clade support was assessed by 1000 bootstrap pseudoreplicates.

The genealogical relationships in nDNA markers (*MC1R*, *NT3*, *PRLR*, *RAG1*) were separately assessed with haplotype (allele) networks. Sequences with more than one heterozygous site were resolved in PHASE 2.1.1^78^ for which the input data were prepared in SeqPHASE^79^. PHASE was run under default settings except for the probability threshold, which was set to 0.7. Allele networks of both analysed markers were examined and drawn using PopArt (http://popart.otago.ac.nz) and the implemented parsimony network algorithm of TCS^80^, with 95% connection limit.

DnaSP 6.00^73^ was used to estimate the number of haplotypes (*h*) and nucleotide diversity (*π*), and uncorrected *p* distances for clades in particular mitochondrial genes or whole mitogenomes.

#### Phylogenetic analysis of mitogenomes

Sequences were aligned using the multiple sequence alignment program Muscle 3.8.31^81^. All gaps and poorly aligned positions were manually removed from the alignment. The total length of the quality trimmed alignment used for phylogenetic inference was 16,756 bp. PartitionFinder 2.1.1^76^ was used to find the best partition scheme for Bayesian phylogenetic inference (BI) with MrBayes 3.2.6^74^. The protein-coding genes were subdivided by codon position. The greedy searching algorithm^76^ and Bayesian information criterion (BIC) were employed to find the best partition scheme for a range of substitution models implemented in MrBayes. The best partition scheme found by PartitionFinder consisted of eight partitions (Table S4). A Bayesian analysis based on the dataset (presented in Table S5) was run in MrBayes as follows: two simultaneous runs with four Markov chains each with 4 × 10^6^ generations and sampling frequency every 500 generations. The first 25% of generations were discarded as burn-in. Convergence of runs was assessed by examination of the average standard deviation of split frequencies and the potential scale reduction factor. Stationarity was confirmed by examining posterior probability, log-likelihood, and all model parameters by the effective sample sizes (obtained ESSs were greater than 800) in the program Tracer 1.7.1^77^. The best partition scheme for maximum likelihood (ML) analysis and the ML search were carried out in IQ-TREE 2.1.2^82^. The ModelFinder^83^ macros in IQ-TREE were used to choose the optimal partitioning scheme using BIC for a range of substitution models implemented in IQ-TREE. The best partition scheme found consisted of six partitions (Table S4). Node support was evaluated with 1000 bootstrap replicates.

#### Principal component analyses of mitochondrial DNA

Principal Component Analyses (PCAs) of mtDNA were carried out using nine sequences in Cyt *b* dataset, and four sequences of full mitogenomes of the *E*. *quatuorlineata* group as defined in the above-mentioned phylogenetic analysis. The PCAs were carried in the package Adegenet^84^ implemented in the R statistical environment^85^.

#### Divergence dating

When we initially tested molecular clock analysis using BEAST 1.10.4^86^, we revealed the alternative topology of *E. quatuorlineata* as a sister lineage to the group of *E*. *sauromates, E*. *urartica*, and the new clade. This topology was also recovered in the testing analysis of Cyt *b* alone but had no statistical support in all cases. In below described analyses we thus forced the split between *E. quatuorlineata* and *E. sauromates* to be monophyletic following the results of the concatenated phylogeny.

The divergence times using full mitogenomes (Table S5) were estimated using BEAST 1.10.4^86^. The GTR + I + G substitution model was selected in jModelTest2^87^ using the BIC. The analysis was run for 2 × 10^7^ generations with a sampling frequency of 1000 generations, from which 25% were discarded as burn-in. A relaxed uncorrelated lognormal clock model, birth–death model of speciation^88^, and random starting tree were applied. The analysis was repeated four times and parameter log files and the phylogenetic trees were combined using LogCombiner 1.10.4. To assess the convergence and effective sample sizes (for ESSs > 200) for all parameters, we used Tracer 1.7.1^77^. The final phylogenetic tree was calculated in TreeAnnotator 1.10.4. The phylogenetic trees were visualized using FigTree 1.4.4 software^89^. We used the following calibration points: the split between *Pantherophis* and *Pituophis*: a mean date of 15.5 Mya (9.5–25.3 Mya; lognormal distribution) normal distribution based on the oldest known rat-snake, *Pantherophis kansensis* (Gilmore, 1938) from the early Barstovian of the Miocene^90^; the divergence between the genera *Elaphe* and *Orthriophis* was placed at 19.7 Mya (15.6–23.8 Mya; normal distribution) as previously estimated in a time-calibrated phylogeny using five fossil calibrations^91^.

The divergence times using Cyt *b* (Table S5) were estimated using the same approach with the following differences compared to the mitogenome-based analysis: HKY + I + G substitution model, analysis was run for 1 × 10^7^ generations, sampling frequency 1000 generations, 25% were discarded as burn-in. We used four ‘external’ calibration points previously established and used in divergence dating of Colubridae^90–93^: (a) the MRCA of the Lampropeltini was assigned a mean date of 20.6 Mya (lognormal 95% CI 11.4–37.1 Mya); (b) the divergence between *Pantherophis* and *Pituophis* was assigned a mean date of 15.5 Mya (lognormal 95% CI 9.5–25.3 Mya); (c) the divergence between the genera *Cemophora* and *Lampropeltis* was assigned a mean date of 13.75 Mya (lognormal 95% CI 8.4–24.4 Ma); (d) the divergence between *Lampropeltis getula* and *Stilosoma extenuatum* was assigned a mean date of 6.8 Mya (lognormal 95% CI 4.75–9.94 Mya). See^90^ for a detailed explanation of the selection of these calibrations.

### Species delimitation

To support hypothesis about divergent clades in the *E*. *quatuorlineata* group, we used a Bayesian implementation of the Poisson tree processes model (bPTP^94^; https://species.h-its.org/) for the species delimitation. For these purposes, we used the concatenated dataset used for ML and BI analysis (see above) and the Newick format of tree from BI analysis (MrBayes 3.2.6^74^) following default settings of the analysis.

### Morphology

#### Meristic characters, morphometry, and coloration

We examined morphological data from 22 specimens of *Elaphe* from the Southern Levant. We recorded 19 meristic characters: preoculare; postoculare; temporale; posttemporale; supralabialia; supralabiale contacting the eyes; sublabiale; gulars in transverse rows between the last two sublabiale; gulars touching anterior inframaxillares; gulars between posterior inframaxillares; dorsalia + temporalia touching parietals; preventrale; ventralia; dorsalia at forebody; dorsalia at midbody; dorsalia at hindbody; subcaudalia; anale; and dorsals in the line from end of the head to cloaca. We also measured 15 distances (to the nearest 0.01 mm): rostrum height; rostrum width; inter-nostril distance; loreal length; eyes diameter; head length; head width; head height; supraoculare width; frontale width; frontale length; anterior inframaxillare length; posterior inframaxillare length; snout-vent length; and tail length (see^25^, Table S2) for description of these characters. Meristic characters were obtained using a stereomicroscope. Measurements were taken with digital calipers (± 0.01 mm) and a measurement tape (to the nearest 0.01 mm for body and tail lengths). A list of examined specimens is presented in Table S1 and a list of examined characters in Table 2 following^4,25^. Coloration in life was described based on specimens observed during field trips to Lebanon and in a specimen kept in captivity at the Zoological Research Garden of Tel Aviv University. Coloration in preservative was based on vouchers deposited at the Steinhardt Museum of Natural History, Tel Aviv University (TAU). Description format of the new species follows^25^.

#### Hemipenes description

We prepared the hemipenis of one specimen of *Elaphe* from the Israeli-controlled part of Mount Hermon (TAU-R 13149) following the protocols of^95–97^. Terminology for hemipenis description and comparisons with hemipenal morphology of *E. quatuorlineata* and *E. sauromates* were based on descriptions provided by^39^.

### Ecology

#### Species distribution modelling

Using MaxEnt 3.3.3^98^, we modelled the potential distribution of the *Elaphe* population from the Southern Levant. We used 40 unique georeferenced presence localities (Table S1). We downloaded and fitted 23 bioclimatic and four landscape layers (contrast, elevation, evenness, slope) from the CHELSA database (https://chelsa-climate.org/). We used ENMTools 1.3^99^ to filter occurenceds and exclude correlated variables. We retained all variables with intercorrelations lower than 0.75 and variables considered to be ecologically important. Model performance was assessed using AUC and estimated the relative contribution of each variable to the model. The spatial resolution was 30 arc seconds. The final dataset contained 13 bioclimatic variables: Bio1, Bio6, Bio8-10, Bio12-19 (https://www.worldclim.org/data/bioclim.html), and four landscape variables.

### Taxonomy

#### Nomenclatural act

The electronic version of this article in portable document format will represent a published work according to the International Code of Zoological Nomenclature (ICZN), and hence the new name contained in the electronic version is effectively published under that Code from the electronic edition alone. This published work and the nomenclatural act it contains have been registered in ZooBank (http://zoobank.org), the online registration system for the ICZN. The ZooBank Life Science Identifier (LSID) for this publication is: urn:lsid:zoobank.org:pub:00EA160D-3EE6-4AD6-8635-0ABEB9499748. The electronic edition of this paper was published in a journal with an ISSN and has been archived and is available from PubMed Central.

## Supplementary Information

Supplementary Information.

## Data Availability

DNA sequences used in the present study are deposited in GenBank. Supporting Information files are available in the online version of this article.
