# Supplementary material for: A new, rare, small-ranged, and endangered mountain snake of the genus Elaphe from the Southern Levant
Source: Sci Rep. 2023 Mar 24;13:4839. doi: 10.1038/s41598-023-30878-4 (PMC10038995; doi:10.1038/s41598-023-30878-4)
Supplement: Supplementary file 1 — Supplementary Information. [file 41598_2023_30878_MOESM1_ESM.pdf]

scientific reports

Supplementary Information for

A new, rare, small-ranged, and endangered mountain snake of the genus *Elaphe* from the Southern Levant

Daniel Jablonski, Marco Antônio Ribeiro-Júnior, Evgeniy Simonov, Katarína Šoltys, Shai Meiri

Contents

Appendix 1 2
Table S1 3
Table S2 6
Table S3 9
Table S4 11
Table S5 12
Figure S1 13
Figure S2 14
Figure S3 15
Figure S4 16
Figure S5 17
Figure S6 18
Figure S7 19
Figure S8 20
Figure S9 21
Figure S10 22
Figure S11 23
Figure S12 24
Figure S13 25
Figure S14 26
Figure S15 27
References 28

Appendix 1: Abstracts of the study in Hebrew and Arabic.

Abstract in Hebrew

תקציר

הסוג *Elaphe* Fitzinger, 1833 כולל 17 מינים של נחשים ארואסיאתיים, כריזמטיים, גדולים, ולא ארסיים. במערב תחום תפוצתו הסוג מיוצג על ידי שלושה מינים בקבוצת ה-*Elaphe quatuorlineata*, הנפוצים ממרכז אסיה ועד איטליה. גבול התפוצה הדרומי של הסוג מצוי בדרום הלבאנט, והאוכלוסיה שם (בדרום סוריה ולבנון וצפון מזרח ישראל) מופרדת מאוכלוסיות אחרות בסוג בפער של כ-400 ק"מ. אוכלוסיה זו ידועה רק מאמצע שנות ה-60 של המאה ה-20, וכיוון שהנחשים בה נדירים ביותר, היא כמעט לא נחקרה. בעבודתנו בחנו את השונות המורפולוגית והגנטית של נחשים מאוכלוסיה זו בהתבסס על נתונים מוזיאוניים, על תצפיותינו בשדה ועל נתוני ספרות על פני כל תחום תפוצתם בסוריה, לבנון וישראל. בדקנו סמנים מיטוכונדריאלים וגרעיניים וכן גנומים מיטוכונדריאלים שלמים של שלושה המינים המוכרים בקבוצה, ושל אוכלוסיית דרום הלבאנט. מצאנו, עם תמיכה סטטיסטת חזקה, ארבע שושלות שהתפצלו זו מזו לפני זמן רב. שלוש מהן מייצגות את המינים המוכרים. אנו מתארים כאן את השושלת הרביעית, שנבדלת גנטית ומורפולוגיות מכל המינים המתוארים בסוג, כמין חדש: *Elaphe druzei* **sp. nov.** קבוצה זו התפצלה לראשונה משושלות אחרות בסוג במיוקן המאוחר, לפני כ 5.1—3.9 מיליון שנה. הדגם הביוגיאוגרפי שמצאנו תומך בסברה כי הלבאנט הוא מרכז עיקרי של מגוון ביולוגי ואנדמיזם בממלכה הפליארקטית. המין החדש הוא אחד הנדירים בנחשי האיזור, ובצרוף עם תחום תפוצתו הקטן יש צורך בפעולה אקטיבית על מנת לשמרו, שאנו מקווים שתביא לשיתוף פעולה מחוקרים וגופי שמירת טבע בכל המדינות בהן הוא מצוי.

Abstract in Arabic

الملخص

يشتمل جنس *Elaphe* Fitzinger, 1833 على 17 نوع من الثعابين غير السامة الكبيرة والبديعة منتشرة في العالم القديم. في المناطق الغربية من شمال العالم القديم يتمثل هذا الجنس بثلاثة أنواع من مجموعة *Elaphe quatuorlineata* تنتشر بشكل واسع يمتد من شبه الجزيرة الإيطالية الى آسيا الوسطى. أبعد جماعة جنوبا هي من هذا الجنس منتشرة على جبال شرق المتوسط تفصلها عن باقي جماعات هذا الجنس قرابة 400 كلم. هذه الجماعة معروفة منذ حوالي 50 سنة في المراجع العلمية ولكن لم تجري دراستها بسبب ندرتها الشديدة. لقد قمنا بدراسة هذه الجماعة من الناحية الشكلية والجينية من المعلومات المتوفرة عموما ومن ما لدينا شخصيا وما في المراجع العلمية المنشورة للمناطق الثلاثة حيث توجد تلك الثعابين وهي مناطق الجولان تحت الاحتلال الاسرائيلي ولبنان وسورية. معتمدين على 9 جينات نووية وميتكوندرية وسلاسل جينية ميتوكوندرية كاملة تشمل ثلاثة أصانيف متعارف عليها حاليا وهي *E. quatuorlineata, E. sauromates,* *E. urartica* وثعابين من جبال شرق المتوسط، تبين لنا وجود اربعة فروع حيوية مدعومة إحصائيا. ثلاثة من هذه الفروع الحيوية تنتمي الى الأنواع الثلاثة المتعارف عليها لكن الفرع الرابع المتميز شكلا عن باقي الأنواع في هذا الجنس، ينتمي الى نوع جديد هو. *Elaphe druzei* **sp. nov** نقدم وصفا له هنا. يقدر وقت حدوث التفرع الأساسي لهذا النوع أثناء أواخر العصر الحديث الوسيط (الميوسيني) مع تشعبات لاحقة منذ 5.1 و 3.9 مليون سنة. ان نسق الإنتشار الجغرافي الحيوي، وما ظهر معنا من نتائج، يدعم أهمية منطقة الشام كمصدر أساسي لظاهرة التوطن (endemism) والتنوع الحبوي في الأوراسيا. هذا النوع الجديد أذاً هو أحد أكثر الأنواع ندرة في المناطق الغربية من شمال العالم القديم. بالنظر الى ندرة هذا النوع وضيق نطاق انتشاره يحتاج هذا النوع الجديد الى حماية مستعجلة ومتشددة بالتعاون بين كل الباحثين والمعنيين في كل مناطق انتشاره.

Table S1. An overview of chronologically ordered museum voucher specimens and observations from literature, citizen science, or own field data on *Elaphe druzei* **sp**. **nov**. from Israel, Lebanon, and Syria.

Museum voucher number ID	Genetic sample ID	country	Locality	Date	N	E	Elevation	age	sex	collector	source
**TAU-R 19438 (paratype)**	-	Israel (Israeli-controlled Golan Heights)	Majdal Shams, few kilometers above, southern slope of Mt. Hermon	1 June 1971	33.279	35.776	~1300	adult	F	Yossi Levari	Zinner (1972)[1]
**TAU-R 19514**	-	Israel (Israeli-controlled Golan Heights)	Hermon, above the upper cable car	18 June 1973	33.305	35.784	~1800	adult	F	Yossi Levari	This study
**TAU-R 19145 (paratype)**	-	Israel (Israeli-controlled Golan Heights)	Hermon	22 June 1973	33.305	35.773	2100	subadult	F	Israeli Defense Forces	This study
**TAU-R 11147**	-	Israel (Israeli-controlled Golan Heights)	Hermon	30 May 1975	33.29	35.78	1800	juvenile	?	unknown	This study
**TAU-R 19144**	-	Israel (Israeli-controlled Golan Heights)	Hermon, 1 km SE to the ski site; on limestone among Quercus trees	11 June 1976	33.306	35.774	~1660	juvenile	?	Shmida Avi	This study
**TAU-R 11463**	-	Israel (Israeli-controlled Golan Heights)	Hermon	24 May 1977	33.27	35.76	~1270	adult	M	A. Pirolherg	This study
**TAU-R 13149**	-	Israel (Israeli-controlled Golan Heights)	Hermon	1981	33.27	35.76	~1270	adult	M	R. Kopon	This study
-	-	Israel (Israeli-controlled Golan Heights)	Hermon, 50 m above the lower cable car station	2 June 1983	~33.31	~35.77	~1700	adult	M	Yehudah L. Werner	Werner (2016: 262 p.)[2]
**TAU-R 13051 (holotype)**	-	Israel (Israeli-controlled Golan Heights)	Majdal Shams, Hermon	18 August 1983	33.27	35.77	1100	adult	F	Yaakov Pessah	This study
**TAU-R 14382**	-	Israel (Israeli-controlled Golan Heights)	Hermon	1985	-	-	-	adult	F?	captivity	This study
**TAU-R 14130**	-	Israel (Israeli-controlled Golan Heights)	Hermon	6 October 1985	33.31	35.8	1900	adult	F	I. Zuri	This study
-	-	Israel (Israeli-controlled Golan Heights)	Hermon	1 June 1987	~33.27	~35.76	~1990	juvenile	?	Yehudah L. Werner	Werner (2016: 263 p.)[2]
**TAU-R 14131**	CUHC 6719	Israel (Israeli-controlled Golan Heights)	Hermon (Zoo exposition)	5 July 1990	~33.27	~35.76	~1300	adult	M	captivity	This study
**TAU-R 17168**	-	Israel (Israeli-controlled Golan Heights)	Hermon Nature Reserve	7 June 1993	33.308	35.773	1400	adult	F	Yosi Vaadia	This study
-	-	Israel (Israeli-controlled Golan Heights)	Hermon, north of Majdal Shams	1998	~33.28	~35.77	~1490	adult	?	Herman A.J. In den Bosch	In den Bosch (1999)[3]
-	-	Israel (Israeli-controlled Golan Heights)	Hermon	1 June 2018	33.302	35.773	~1700	adult	?	citizen science data	https://www.inaturalist.org/observations/13341574

**TAU-R 19070 (paratype)**	CUHC 9363	Israel (Israeli-controlled Golan Heights)	Hermon	16 May 2019	33.292	35.772	1602	adult	M	Eran Levin	This study
-	-	Israel (Israeli-controlled Golan Heights)	Hermon	26 May 2020	~33.302	~35.773	-	adult	?	Ori Fragman-Sapir (citizen science data)	https://www.inaturalist.org/observations/47437675
		Israel (Israeli-controlled Golan Heights)	Hermon	24 May 2022	33.3071	35.7954	1976	adult	F?	Simon Jamison	This study
-	-	Israel (Israeli-controlled Golan Heights)	Hermon	19 July 2022	~33.31	~35.8	-	juvenile	?	citizen science data	https://www.instagram.com/p/CfOF4YDq1x9/?igshid=NDc0ODY0MjQ%3D
-	-	Lebanon	Barouk, Maaser Al Shouf	?	33.6833	35.70	-	?	?	unknown	Hraoui-Bloquet et al. (2002) [4]
-	-	Lebanon	Niha Cedars, Shouf	?	33.5942	35.6294	1400	?	?	unknown	Hraoui-Bloquet et al. (2002) [4]
**AUB 2**	-	Lebanon	Barouk, Kfar Slouan	?	33.859	35.783	1420	adult	F	unknown	This study
-	-	Lebanon	Barouk, Maaser Al Shouf	1995 or 1997	33.60	35.65	1850	juvenile	?	unknown	In den Bosch et al. (1998) [5]
**AR-0881 (AUB 1)**	-	Lebanon	Kfar Selwane	20 April 2006	33.859	35.783	1420	adult	M	Ramzi Maghvabi	This study
-	-	Lebanon	Shouf/Barouk	3 May 2013	33.8599	35.7836	1387	adult	?	citizen science data	This study
-	-	Lebanon	Rachaiya	2015-2022	33.486	35.86	1235	adult	?	citizen science data	This study
-	-	Lebanon	Yammouneh	13 June 2016	34.1044	36.0115	1540	adult	?	citizen science data	This study
-	CUHC 6791	Lebanon	Barouk, Kfar Slouan	1 April 2018	33.859	35.783	1420	adult	F	Daniel Jablonski, Riyad Sadek	This study
-	-	Lebanon	Zaarour	5 May 2018	33.9209	35.8114	1655	adult	?	citizen science data	This study
-	-	Lebanon	Zaarour	15 May 2018	33.9209	35.8115	1655	juvenile	?	citizen science data	This study
-	-	Lebanon	Zaarour	8 June 2018	33.8599	35.7836	1387	adult	?	citizen science data	This study
-	-	Lebanon	Majdal Tarchich	10 June 2018	33.8959	35.8024	1531	adult	?	citizen science data	This study
-	-	Lebanon	Tarchich	12 June 2018	33.8720	35.8199	1638	adult	?	citizen science data	This study
-	-	Lebanon	Yammouneh	26 September 2018	34.1044	36.0115	1540	juvenile	?	citizen science data	This study
-	-	Lebanon	Shouf/Ain Zhalta	15 May 2019	33.7480	35.7274	1379	adult	?	citizen science data	This study
-	-	Lebanon	Zaarour	16 May 2019	33.91543	35.81650	1850	adult	?	citizen science data	This study
-	CUHC 11712	Lebanon	Zaarour	3 June 2019	33.92098	35.81147	1655	adult	F	Nader Zeitouni (citizen science data)	https://www.inaturalist.org/observations/46789062
-	-	Lebanon	Yammouneh	10 June 2019	34.10444	36.01157	1540	adult	?	citizen science data	This study
-	-	Lebanon	Shouf/Barouk	30 June 2019	33.85996	35.78365	1387	adult	?	citizen science data	This study
-	-	Lebanon	Zaarour	13 May 2020	33.91106	35.79814	1705	adult	?	citizen science data	This study

-	-	Lebanon	Shouf/Barouk	28 May 2020	33.85996	35.78365	1387	adult	?	citizen science data	This study
-	-	Lebanon	Zaarour	9 June 2020	33.92098	35.81147	1655	adult	?	citizen science data	This study
-	-	Lebanon	Touaiti	2 May 2021	33.86799	35.85188	1554	adult	?	Rachad Sakr (citizen science data)	https://www.inaturalist.org/observations/77491107
**NMW 23472**	-	Syria	Quneitra, Camp Fauar, Golan Heights	1976	33.15972	35.84166	948	adult	F	Karl Kollnberger	Tiedemann & Häupl (1978) [6]
-	-	Syria	4 km W Hadar	?	33.28055	35.82638	1240	?	?	unknown	Esterbauer (1992) [7]
-	-	Syria	2 km S Halas	?	33.24861	35.94583	1000	?	?	unknown	Esterbauer (1992) [7]
-	-	Syria	Halboun, 30 km NW Damascus	22 May 2020	33.66562	36.24649	2200	adult	?	Ahmad Qawi	Qawi et al. (2019) [8]

Abbreviations: AUB = American University of Beirut; CUHC = Comenius University Herpetological Collection; NMW = Naturhistorisches Museum Wien (Natural History Museum Vienna); TAU = Tel Aviv University.

Table S2. A list of genetic material and sequences used for the concatenated molecular phylogeny trees and nuclear allele networks only* (*Elaphe quatuorlineata* group). na = not available; - available, not used.

**Genetic sample ID**	**In-group species**	***16S***	***COI***	***ND4***	**Cyt *b***	***Rag1***	***MC1R***	***C-mos***	***NT3***	***PRLR***
**1509**	*Elaphe quatuorlineata*	MK334307 (mitogenome)	MK334307 (mitogenome)	MK334307 (mitogenome)	MK334307 (mitogenome)	MK640344	MK640325	na	OP642478	MK640300
**1124** Holotype: ZDEU 26/2012	*Elaphe urartica*	OP613267 (mitogenome)	MK640299	MK640402	OP613267 (mitogenome)	OP642474	OP642470	MK640324	OP642479	MK640311
**3089**	*Elaphe urartica*	na	-	-	na	MK640359*	MK640340*	na	na	MK640312*
**3090**	*Elaphe urartica*	na	-	-	na	na	na	na	na	MK640313*
**3095**	*Elaphe urartica*	na	-	-	na	MK640360*	na	na	na	MK640314*
**3096**	*Elaphe urartica*	na	-	-	na	MK640361*	na	na	na	MK640315*
**3195**	*Elaphe urartica*	na	-	-	na	MK640362*	MK640341*	na	na	MK640316*
**3196**	*Elaphe urartica*	na	-	-	na	na	na	na	na	MK640317*
**3197**	*Elaphe urartica*	na	-	-	na	na	na	na	na	MK640318*
**3408**	*Elaphe urartica*	na	-	-	na	MK640363*	MK640342*	na	na	na
**3655**	*Elaphe urartica*	na	-	-	na	MK640365*	MK640343*	na	na	na
**278**	*Elaphe sauromates*	na	-	-	na	MK640345*	MK640326*	MK640319*	na	MK640301*
**1125**	*Elaphe sauromates*	na	-	-	na	na	MK640327*	MK640320*	na	MK640302*
**1126**	*Elaphe sauromates*	na	-	-	na	na	MK640328*	MK640321*	na	MK640303*
**1176**	*Elaphe sauromates*	na	-	-	na	MK640346*	MK640329*	na	na	na
**1177**	*Elaphe sauromates*	na	-	-	na	MK640347*	MK640330*	na	na	na

**1179**	*Elaphe sauromates*	MK070315 (mitogenome)	MK640288	MK640377	MK070315 (mitogenome)	MK640348	MK640331	MK640322	na	MK640304
**2375**	*Elaphe sauromates*	na	-	-	na	na	MK640332*	MK640323*	na	MK640305*
**2376**	*Elaphe sauromates*	na	-	-	na	na	MK640333*	na	na	MK640306*
**2378**	*Elaphe sauromates*	na	-	-	na	MK640349*	MK640334*	na	na	na
**2494**	*Elaphe sauromates*	na	-	-	na	na	MK640335*	na	na	MK640307*
**2892**	*Elaphe sauromates*	na	-	-	na	MK640352*	MK640336*	na	na	MK640308*
**2893**	*Elaphe sauromates*	na	-	-	na	MK640353*	na	na	na	MK640309*
**3193**	*Elaphe sauromates*	na	-	-	na	MK640354*	MK640337*	na	na	na
**3194**	*Elaphe sauromates*	na	-	-	na	MK640355*	na	na	na	MK640310*
**3406**	*Elaphe sauromates*	na	-	-	na	MK640356*	MK640338*	na	na	na
**3447**	*Elaphe sauromates*	na	-	-	na	MK640358*	MK640339*	na	na	na
**6719**	*Elaphe druzei* **sp. nov**.	OP613266 (mitogenome)	OP618085	OP642464	OP613266 (mitogenome)	OP642475	OP642471	na	OP642480	na
**6791**	*Elaphe druzei* **sp. nov**.	OP616942	OP618086	OP642465	OP642463	OP642476	OP642472	na	OP642481	OP642468
**9363** Paratype: TAU-R 19070	*Elaphe druzei* **sp. nov**.	OP616943	OP618087	OP642466	OP642462	OP642477	OP642473	na	OP642482	OP642469
**11712**	*Elaphe druzei* **sp. nov**.	na	na	OP642467	na	na	na	na	na	na
	**Outgroup species**									
	Elaphe anomala	KP900218 (mitogenome)	KP900218 (mitogenome)	KP900218 (mitogenome)	KP900218 (mitogenome)	na	na	na	na	na

Elaphe bimaculata	MK193931	MK064634	DQ902283	MK201283	na	na	DQ902062	na	na
Elaphe cantoris	MK194263	MK064913	DQ902315	MK201564	na	na	DQ902095	na	na
Elaphe carinata	KU180459 (mitogenome)	KU180459 (mitogenome)	KU180459 (mitogenome)	KU180459 (mitogenome)	na	na	JN799415	na	na
Elaphe climacophora	KX277245	AY122688	DQ902285	LC327646	na	na	DQ902064	na	na
Elaphe davidi	KM401547 (mitogenome)	KM401547 (mitogenome)	KM401547 (mitogenome)	KM401547 (mitogenome)	na	na	na	na	na
Elaphe dione	MH460961 (mitogenome)	MH460961 (mitogenome)	MH460961 (mitogenome)	MH460961 (mitogenome)	na	na	KP091853	na	na
Elaphe hodgsonii	MK193983	MK064680	DQ902318	MK201335	na	na	DQ902096	na	na
Elaphe moellendorffi	na	JF700191	DQ902295	DQ902116	na	na	DQ902074	na	na
Elaphe quadrivirgata	AB738958 (mitogenome)	AB738958 (mitogenome)	AB738958 (mitogenome)	AB738958 (mitogenome)	na	na	DQ902078	na	na
Elaphe schrenckii	KP888955 (mitogenome)	KP888955 (mitogenome)	KP888955 (mitogenome)	KP888955 (mitogenome)	na	na	DQ902082	na	na
Elaphe taeniura	KC990021 (mitogenome)	KC990021 (mitogenome)	KC990021 (mitogenome)	KC990021 (mitogenome)	na	na	EF076705	na	na
Elaphe xiphodonta	MZ242100	MZ191164	na	MZ191166	na	na	na	na	na
Elaphe zoigeensis	MK193930	MK064633	na	MK201282	na	na	na	na	na
Euprepiophis mandarinus	MK193941	MK064645	DQ902294	MK201293	na	na	DQ902073	na	na
Gonyosoma frenatum	MW413812 (mitogenome)	MW413812 (mitogenome)	MW413812 (mitogenome)	MW413812 (mitogenome)	na	na	DQ902069	na	na

Table S3. Partition scheme for the concatenated dataset of mitochondrial (*16S*, *COI*, *ND4*, Cyt *b*) and nuclear markers (*Rag1*, *MC1R*, *C-mos, NT3, PRLR*).

RAxML			
Subset	**Best model**	**Partitions names**	**Subset sites**
**1**	GTR+I+G	Gene1_16S_pos1	1-1353
**2**	GTR+G	Gene2_COI_pos1	1354-2225\3
**3**	GTR+G	Gene2_COI_pos2	1355-2225\3
**4**	GTR+G	Gene2_COI_pos3	1356-2225\3
**5**	GTR+G	Gene3_ND4_pos1	2226-3106\3
**6**	GTR+I+G	Gene3_ND4_pos2	2227-3106\3
**7**	GTR+I+G	Gene3_ND4_pos3	2228-3106\3
**8**	GTR+G	Gene4_cytb_pos1	3107-4225\3
**9**	GTR+I+G	Gene4_cytb_pos2	3108-4225\3
**10**	GTR+G	Gene4_cytb_pos3	3109-4225\3
**11**	GTR+G	Gene5_RAG1_pos1	4226-5241\3
**12**	GTR+G	Gene5_RAG1_pos2	4227-5241\3
**13**	GTR+G	Gene5_RAG1_pos3	4228-5241\3
**14**	GTR+G	Gene6_MC1R_pos1	5242-5891\3
**15**	GTR+G	Gene6_MC1R_pos2	5243-5891\3
**16**	GTR+G	Gene6_MC1R_pos3	5244-5891\3
**17**	GTR+G	Gene7_CMOS_pos1	5892-6458\3
**18**	GTR+G	Gene7_CMOS_pos2	5893-6458\3
**19**	GTR+G	Gene7_CMOS_pos3	5894-6458\3
**20**	GTR+G	Gene8_NT3_pos1	6459-7027\3
**21**	GTR+G	Gene8_NT3_pos2	6460-7027\3
**22**	GTR+G	Gene8_NT3_pos3	6461-7027\3
**23**	GTR+G	Gene9_PRLR_pos1	7028-7578\3
**24**	GTR+G	Gene9_PRLR_pos2	7029-7578\3
**25**	GTR+G	Gene9_PRLR_pos3	7030-7578\3

MrBayes			
**1**	GTR+I+G	Gene1_16S_pos1	1-1353
**2**	GTR+G	Gene2_COI_pos1	1354-2225\3
**3**	SYM+G	Gene2_COI_pos2	1355-2225\3
**4**	F81	Gene2_COI_pos3	1356-2225\3
**5**	GTR+G	Gene3_ND4_pos1	2226-3106\3
**6**	HKY+I+G	Gene3_ND4_pos2	2227-3106\3
**7**	HKY+I+G	Gene3_ND4_pos3	2228-3106\3
**8**	GTR+G	Gene4_cytb_pos1	3107-4225\3
**9**	HKY+I+G	Gene4_cytb_pos2	3108-4225\3
**10**	HKY+G	Gene4_cytb_pos3	3109-4225\3
**11**	F81	Gene5_RAG1_pos1	4226-5241\3
**12**	F81	Gene5_RAG1_pos2	4227-5241\3
**13**	JC	Gene5_RAG1_pos3	4228-5241\3
**14**	JC	Gene6_MC1R_pos1	5242-5891\3
**15**	F81	Gene6_MC1R_pos2	5243-5891\3
**16**	F81+I	Gene6_MC1R_pos3	5244-5891\3
**17**	HKY+I	Gene7_CMOS_pos1	5892-6458\3
**18**	K80	Gene7_CMOS_pos2	5893-6458\3
**19**	JC	Gene7_CMOS_pos3	5894-6458\3
**20**	JC	Gene8_NT3_pos1	6459-7027\3
**21**	JC	Gene8_NT3_pos2	6460-7027\3
**22**	JC	Gene8_NT3_pos3	6461-7027\3
**23**	F81	Gene9_PRLR_pos1	7028-7578\3
**24**	K80	Gene9_PRLR_pos2	7029-7578\3
**25**	JC	Gene9_PRLR_pos3	7030-7578\3

Table S4. Partition schemes for Maximum likelihood IQ-TREE and MrBayes analyses based on full mitogenome dataset.

Partition subset	Best model	Partition names
**Maximum likelihood**		
1	TIM2+F+I +G4	tRNA-Phe+12SrRNA+tRNA-Val+16SrRNA+tRNA-Trp+tRNA-Cys+COX1_1+COX2_1+COX3_1+tRNA-Gly+tRNA-Ser2+tRNA-Pro
2	GTR+F+R 2	ND1_1+tRNA-Ile+tRNA-Leu2+tRNA-Gln+tRNA-Met+ND2_1+tRNA-Ala+tRNA-Asn+tRNA-Asp+tRNA-Lys+ATP8_1+ATP6_1+ND3_1+tRNA-Arg+ND4L_1+ND4_1+tRNA-His+tRNA-Leu+ND5_1+ND6_2+ND6_3+tRNA-Glu+CYTB_1+tRNA-Thr
3	HKY+F+R 2	ND1_2+tRNA-Tyr+COX1_2+tRNA-Ser+COX2_2+COX3_2+ND4_2+CYTB_2
4	GTR+F+R 4	ND1_3+ND2_3+COX1_3+COX2_3+ATP8_3+ATP6_3+COX3_3+ND3_3+ND4L_3+ND4_3+ND5_3+ND6_1+CYTB_3
5	TVM+F+R 2	D-loop1+D-loop2
6	TPM3+F+I +G4	ND2_2+ATP8_2+ATP6_2+ND3_2+ND4L_2+ND5_2
**MrBayes**		
1	SYM+I	tRNA-Ser2, tRNA-Phe, COX1_1, COX3_1
2	GTR+I	tRNA-Trp, tRNA-Ile, tRNA-Leu2, tRNA-Ala, tRNA-Gln, tRNA-Glu, tRNA-His, tRNA-Val, CYTB_1, ND1_1, 16SrRNA, 12SrRNA
3	HKY+I	tRNA-Ser, COX1_2, COX2_2, COX3_2, ND1_2
4	GTR+I+G	ND1_3, ND6_1, ND5_3, CYTB_3, ND2_3, ND4_3, ND3_3, ATP8_1, COX2_3, COX3_3, ND4L_1, COX1_3, ATP6_3, ATP8_3
5	HKY+G	tRNA-Arg, tRNA-Gly, tRNA-Pro, D-loop2, D-loop1
6	K80+G	tRNA-Asp, tRNA-Tyr, tRNA-Met, COX2_1, tRNA-Leu, tRNA-Cys, tRNA-Lys, tRNA-Asn, tRNA-Thr
7	GTR+G	ATP8_2, ND6_3, ND6_2, ND4L_1, ND2_1, ATP6_1, ND3_1, ND5_1, ND4_1
8	HKY+I	ND4_2, CYTB_2, ND3_2, ATP6_2, ND4L_1, ND2_2, ND5_2

Table S5. A list of sequences used for tree and divergence dating analyses, separate for full mitogenome and cytochrome *b* datasets.

**Mitogenome**		**Cytochrome *b***	
**species**	**GenBank accession number**	**species**	**GenBank accession number**
Oocatochus rufodorsatus	KC990020	*Liodytes rigida*	AF471052
Pantherophis slowinskii	DQ523162	*Storeria dekayi*	AF471050
Pituophis catenifer	KU833245	*Thamnophis melanogaster*	EF417410
Elaphe taeniura	KC990021	*Pantherophis obsoletus*	AF283643
Elaphe schrenckii	KP888955	*Lampropeltis getula*	AF337089
Elaphe quadrivirgata	AB738958	*Stilosoma extenuatum*	AF337107
Elaphe carinata	KU180459	*Lampropeltis calligaster*	AF337108
Elaphe davidi	KM401547	*Pituophis melanoleucus*	AF337110
Elaphe dione	NC_041068	*Pituophis ruthveni*	AF337111
Elaphe quatuorlineata	MK334307	*Pituophis catenifer*	KU833245
Elaphe sauromates	MK070315	*Lampropeltis mexicana*	AF337146
Elaphe urartica	OP613267	*Lampropeltis triangulum*	AF337168
Elaphe druzei **sp. nov**.	OP613266	*Pantherophis guttatus*	AF337173
		Macroprotodon cucullatus	AF471087
		Cemophora coccinea	AF471091
		Telescopus fallax	AY188039
		Hemorrhois nummifer	AY376742
		Coluber constrictor	AY486913
		Platyceps najadum	AY486919
		Masticophis flagellum	AY486928
		Pantherophis slowinskii	DQ523162
		Pantherophis vulpinus	FJ267654
		Elaphe bimaculata	DQ902104
		Elaphe climacophora	DQ902105
		Elaphe dione	DQ902107
		Elaphe schrenckii	DQ902124
		Zamenis situla	DQ902125
		Senticolis triaspis	DQ902127
		Elaphe quadrivirgata	HQ122362
		Elaphe davidi	KM401547
		Elaphe quatuorlineata	MH444320
		Elaphe quatuorlineata	MH444331
		Elaphe quatuorlineata	MH444337
		Elaphe quatuorlineata	MK334307
		Elaphe quatuorlineata	MH444346
		Elaphe sauromates	MH444358
		Elaphe sauromates	MK070315
		Elaphe urartica	OP613267
		Elaphe druzei **sp. nov**.	OP613266

Figure S1. Phylogenetic tree of the genus *Elaphe* resulting from the ML analysis of the concatenated dataset. Numbers with branches indicate bootstrap support values.

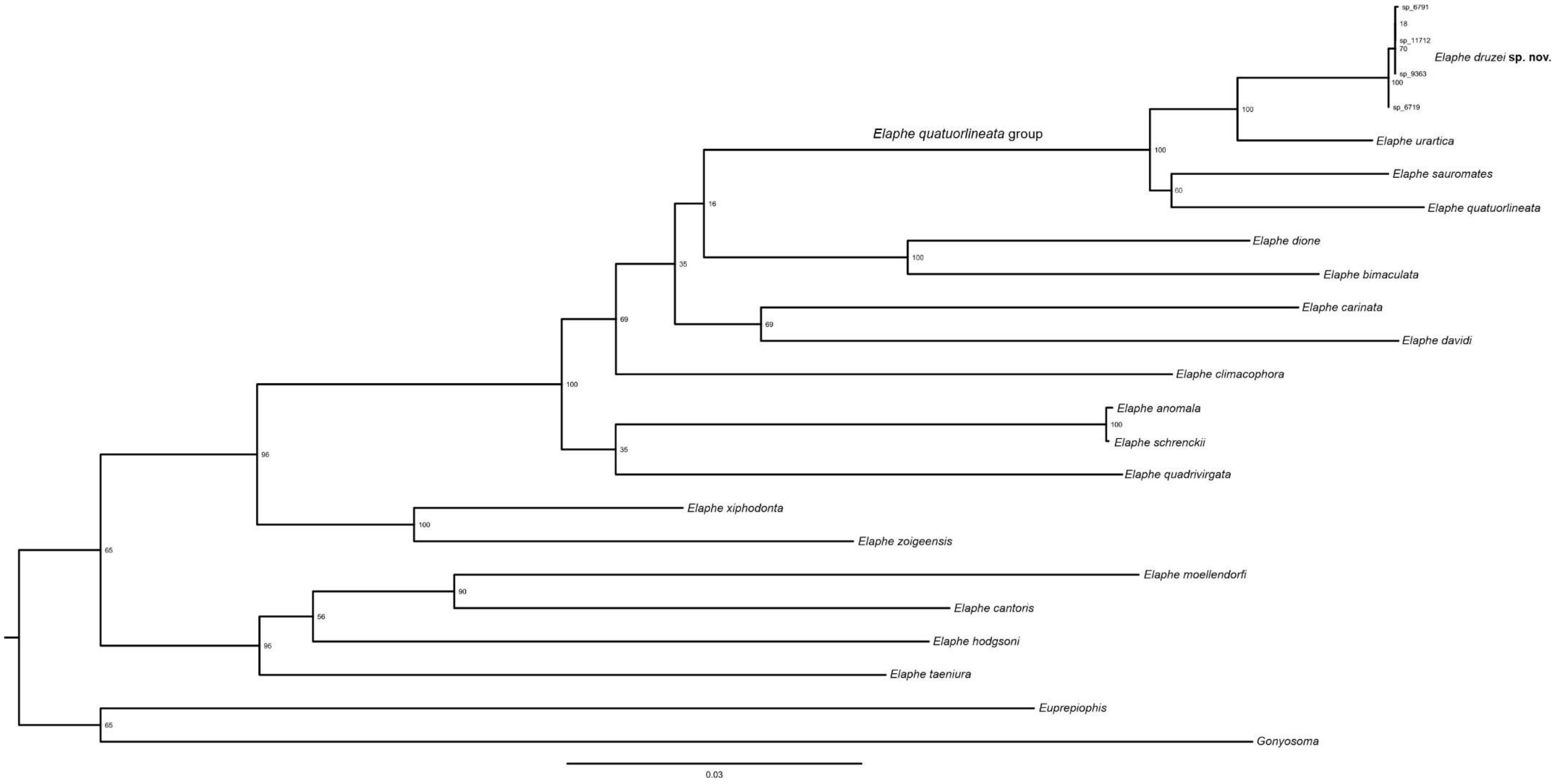

Figure S2. Phylogenetic tree of the genus *Elaphe* resulting from the BI analysis of the concatenated dataset. Numbers with branches indicate Bayesian posterior probabilities support values.

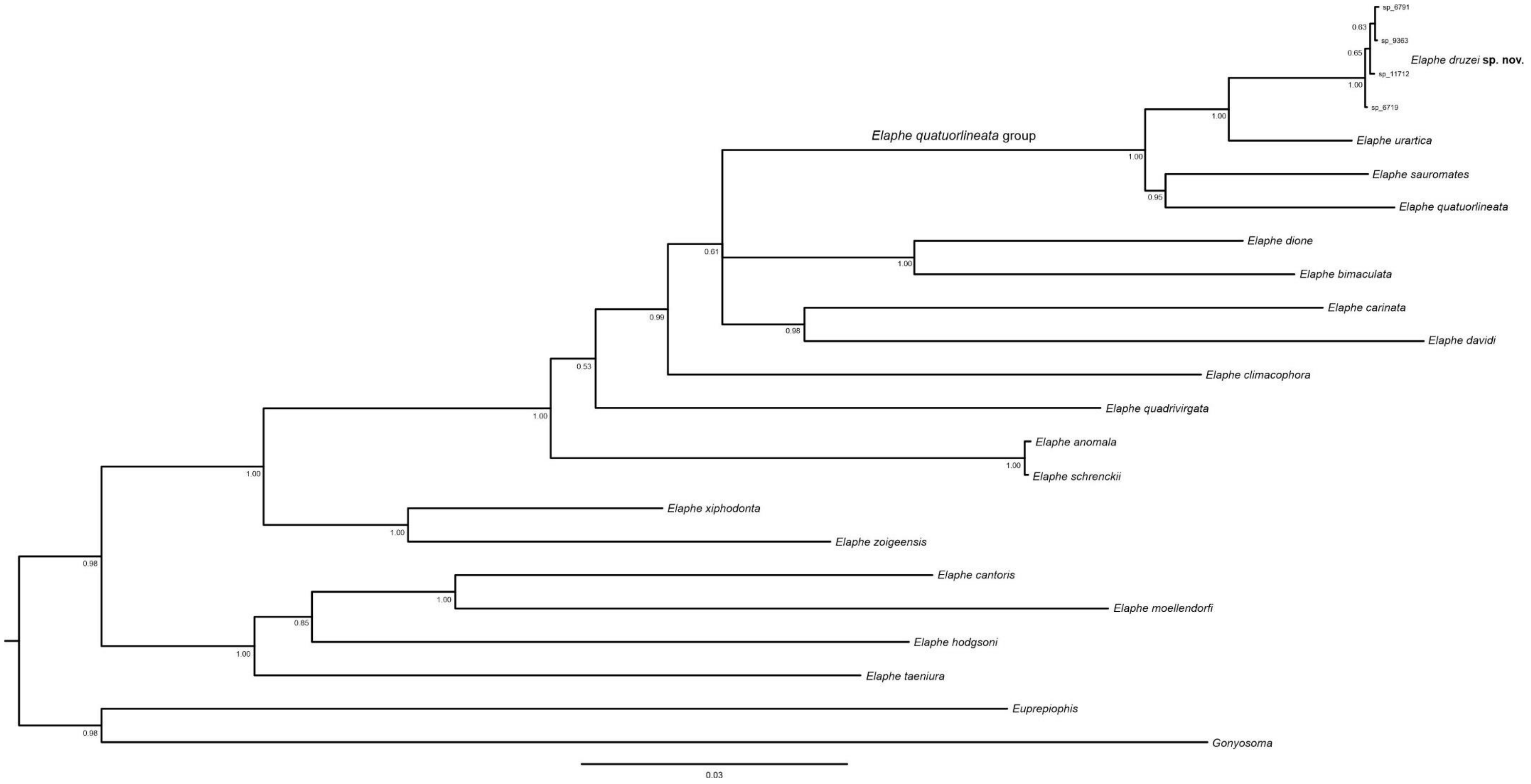

Figure S3. Time-calibrated tree of the mitogenome dataset obtained with BEAST. Numbers above branches indicate mean estimated node ages (in millions of years). The numbers under branches represent Bayesian posterior probabilities support values. The red arrow indicates a constrained node. Blue bars are 95% highest posterior densities of the estimated node ages. The two calibration points are marked with red dots. For the list of sequences see Table S5.

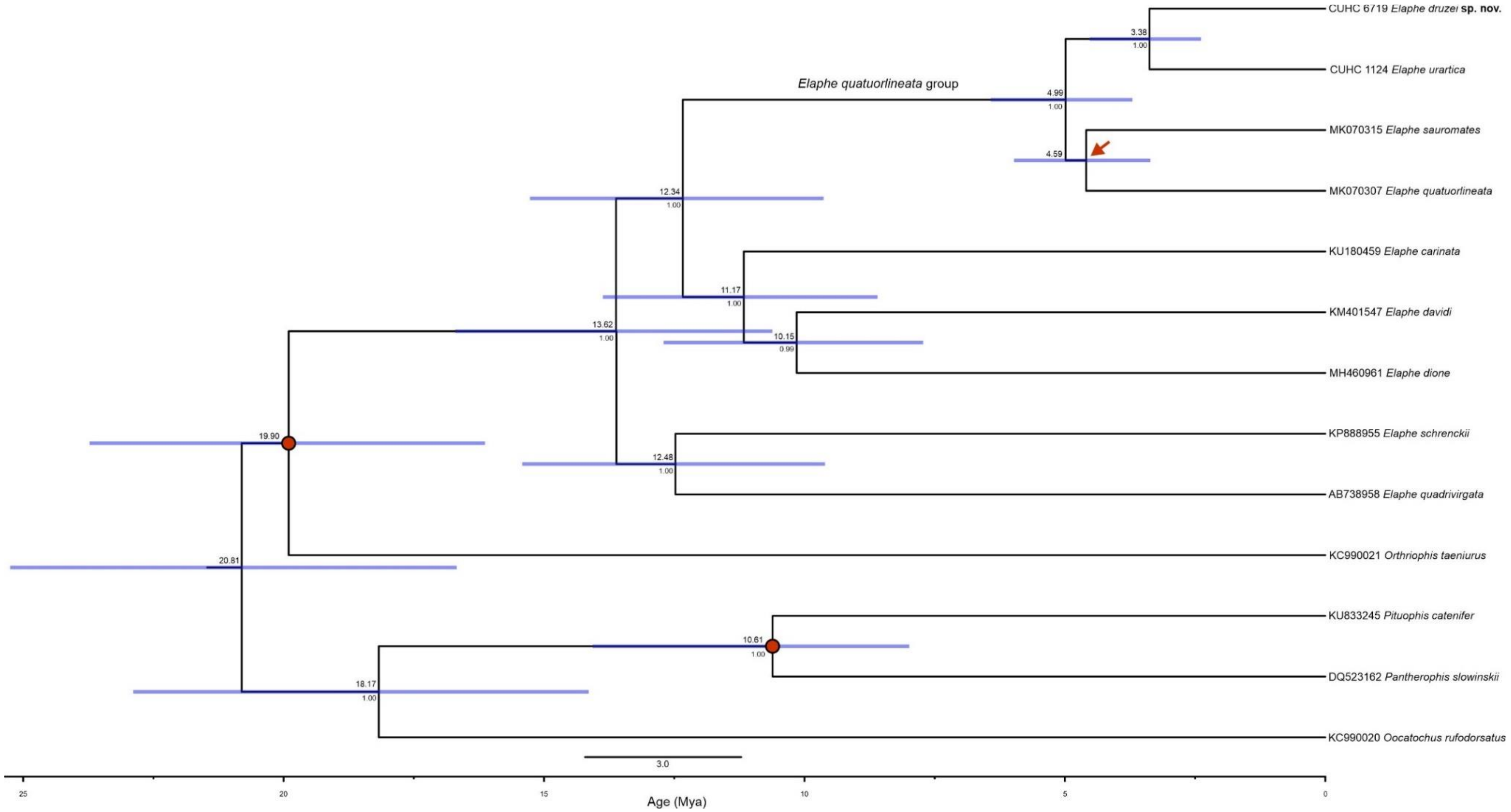

Figure S4. Time-calibrated tree of the cytochrome *b* dataset obtained with BEAST. Numbers above branches indicate mean estimated node ages (in millions of years). The numbers under branches represent Bayesian posterior probabilities support values. The red arrow indicates a constrained node. Blue bars are 95% highest posterior densities of the estimated node ages. The four calibration points are marked with red dots. For the list of sequences see Table S5.

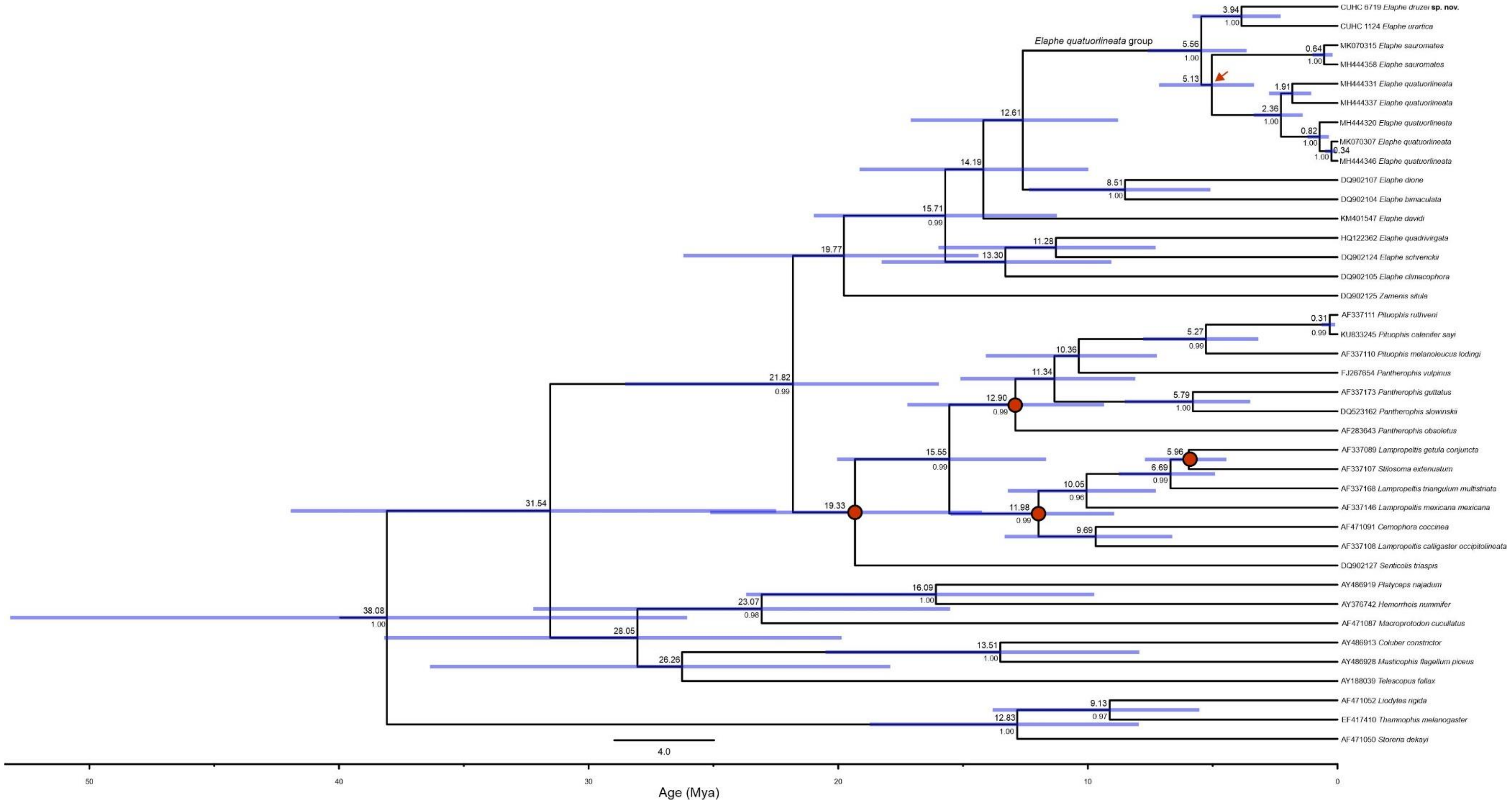

Figure S5. The paratype of *Elaphe druzei* **sp**. **nov**. TAU-R 19070 (adult male) from Hermon, Israel (Israeli-controlled Golan Heights) (16 May 2019). (A-C) dorsal, ventral, and lateral views on the head, (D, E) dorsal and ventral views on the body. Bar = 1 cm. Photographs by Marco Antônio Ribeiro-Júnior.

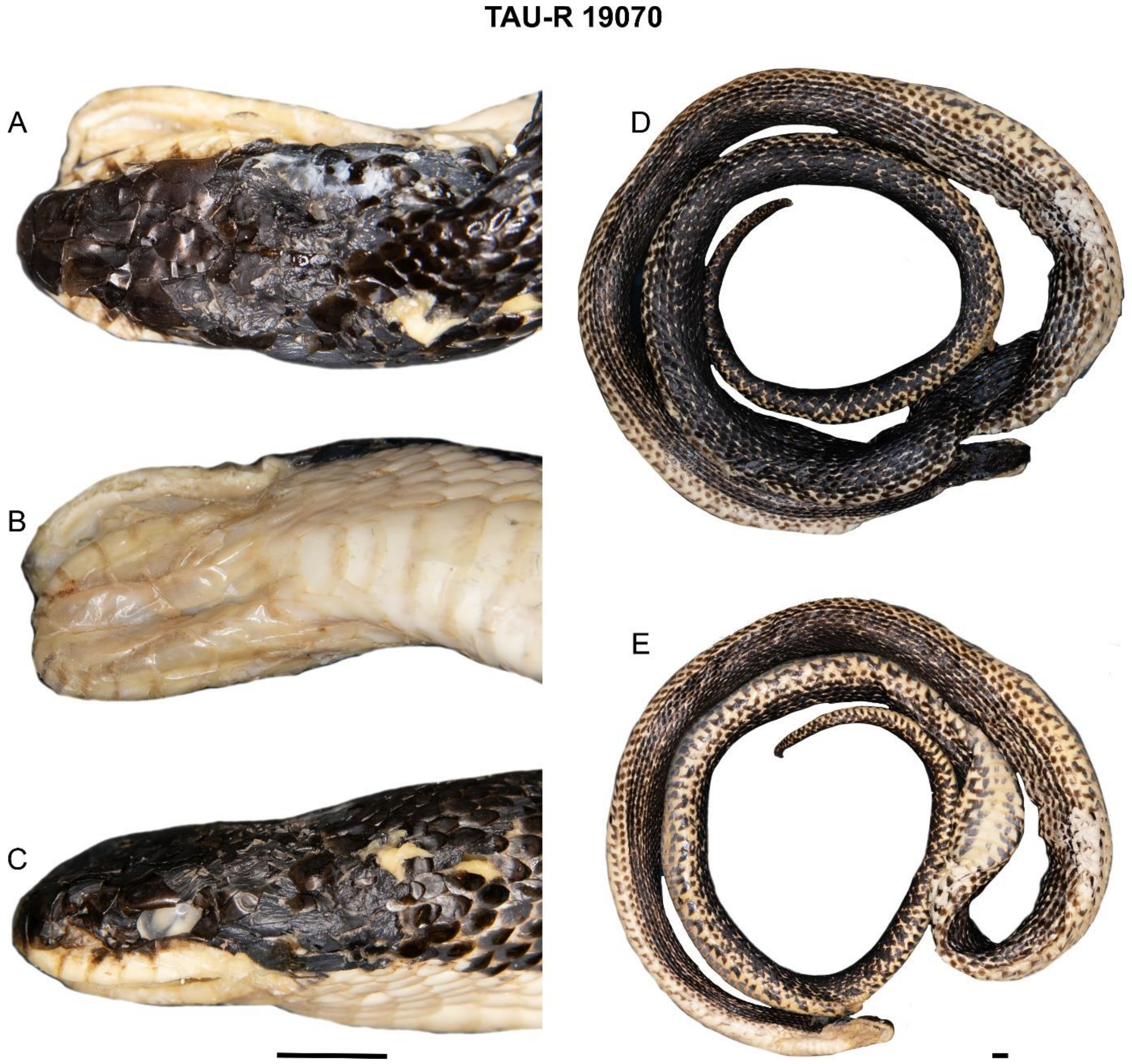

Figure S6. The paratype of *Elaphe druzei* **sp**. **nov**. TAU-R 19145 (adult female) from Hermon, Israel (Israeli-controlled Golan Heights) (22 June 1973). (A-C) dorsal, ventral, and lateral views on the head, (D, E) dorsal and ventral views on the body. Bar = 1 cm. Photographs by Daniel Berkowic.

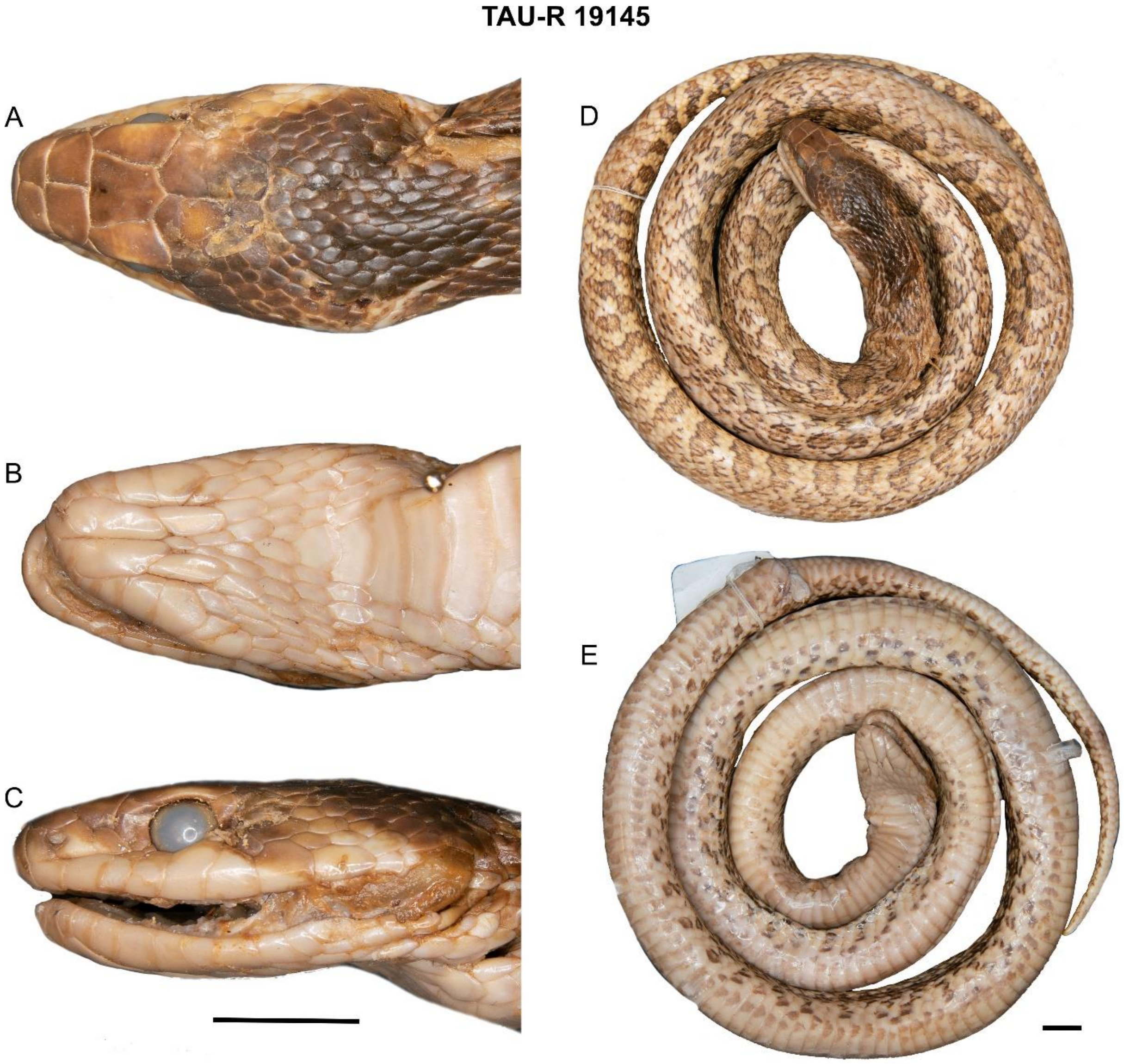

Figure S7. The paratype of *Elaphe druzei* **sp**. **nov**. TAU-R 19438 (adult female) from Hermon, Majdal Shams, southern slope, Israel (Israeli-controlled Golan Heights) (1 June 1971; see [1]). (A-C) dorsal, ventral, and lateral views on the head, (D, E) dorsal, and ventral views on the body. Bar = 1 cm. Photographs by Daniel Berkowic.

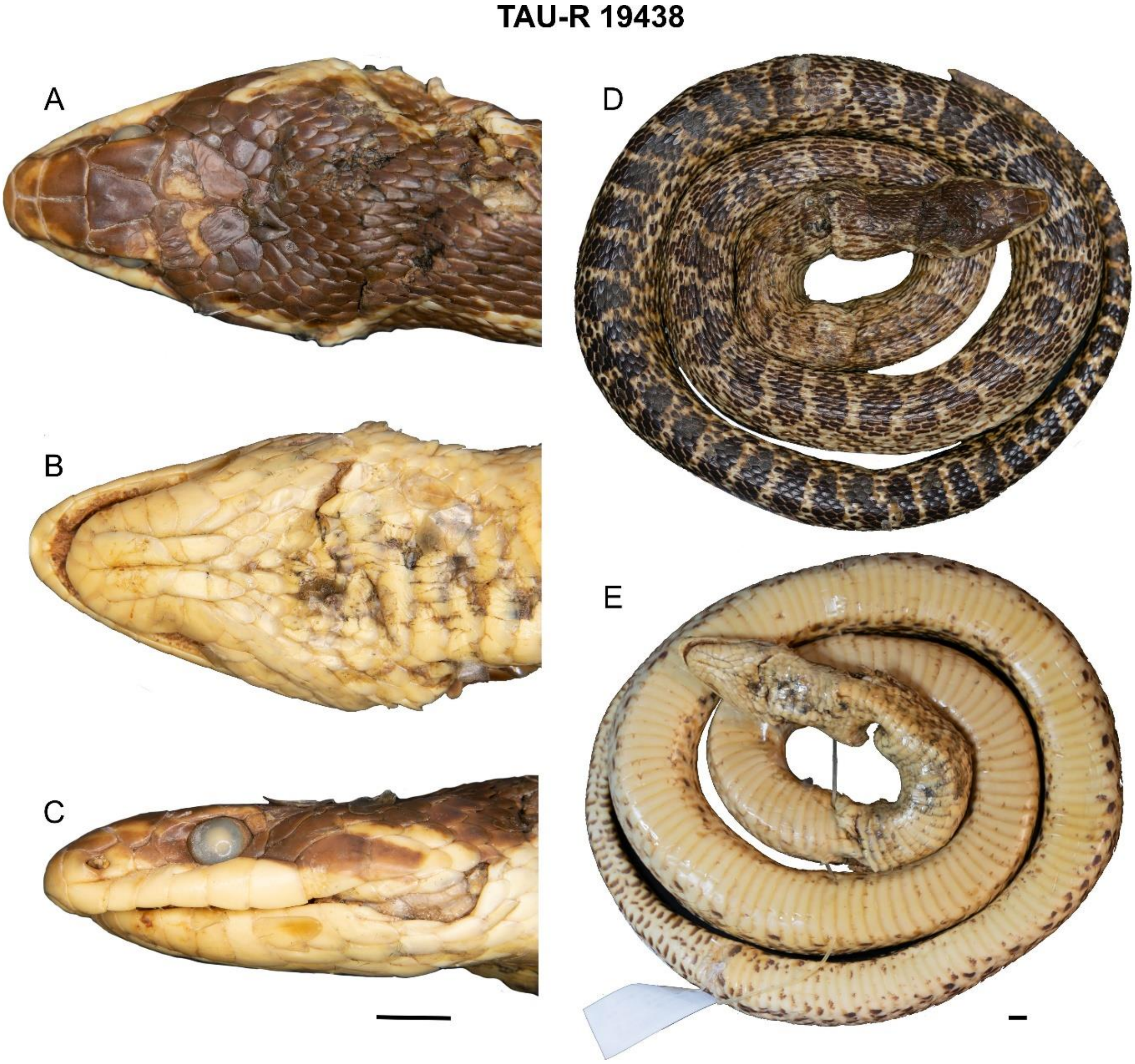

Figure S8. The adult female of *Elaphe druzei* **sp. nov**. (CUHC 6791) from Barouk, Kfar Slouan, Lebanon (1 April 2018). (A-D) dorsal, ventral, and lateral views on the head, (E) dorsal view on the body. Photographs by Daniel Jablonski.

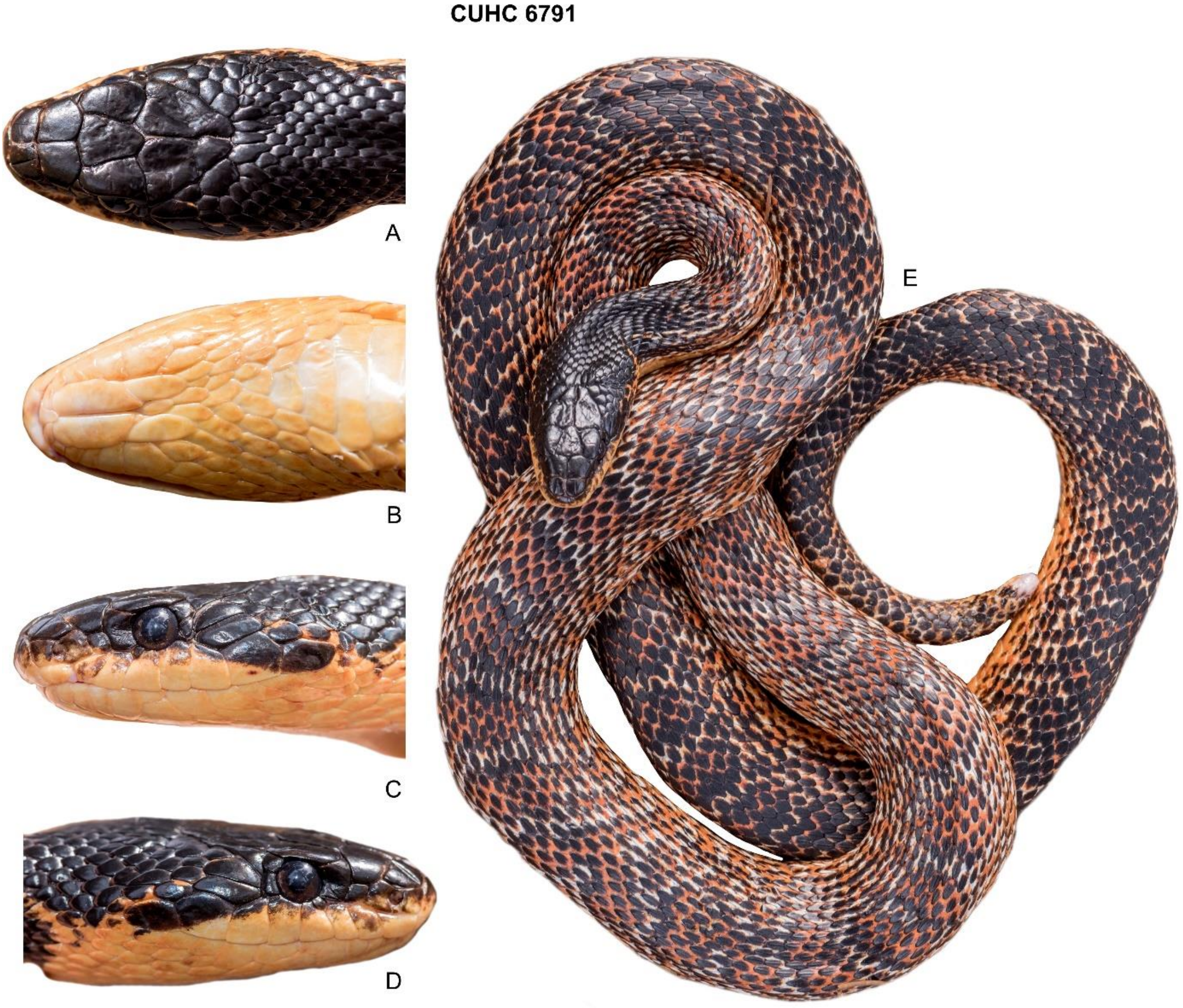

Figure S9. The adult female of *Elaphe druzei* **sp**. **nov**. (CUHC 11712) from Zaarour, Lebanon (May 2022). (A-D) dorsal, ventral, and lateral views on the head, (E) dorsal view on the body. Photographs by Daniel Jablonski.

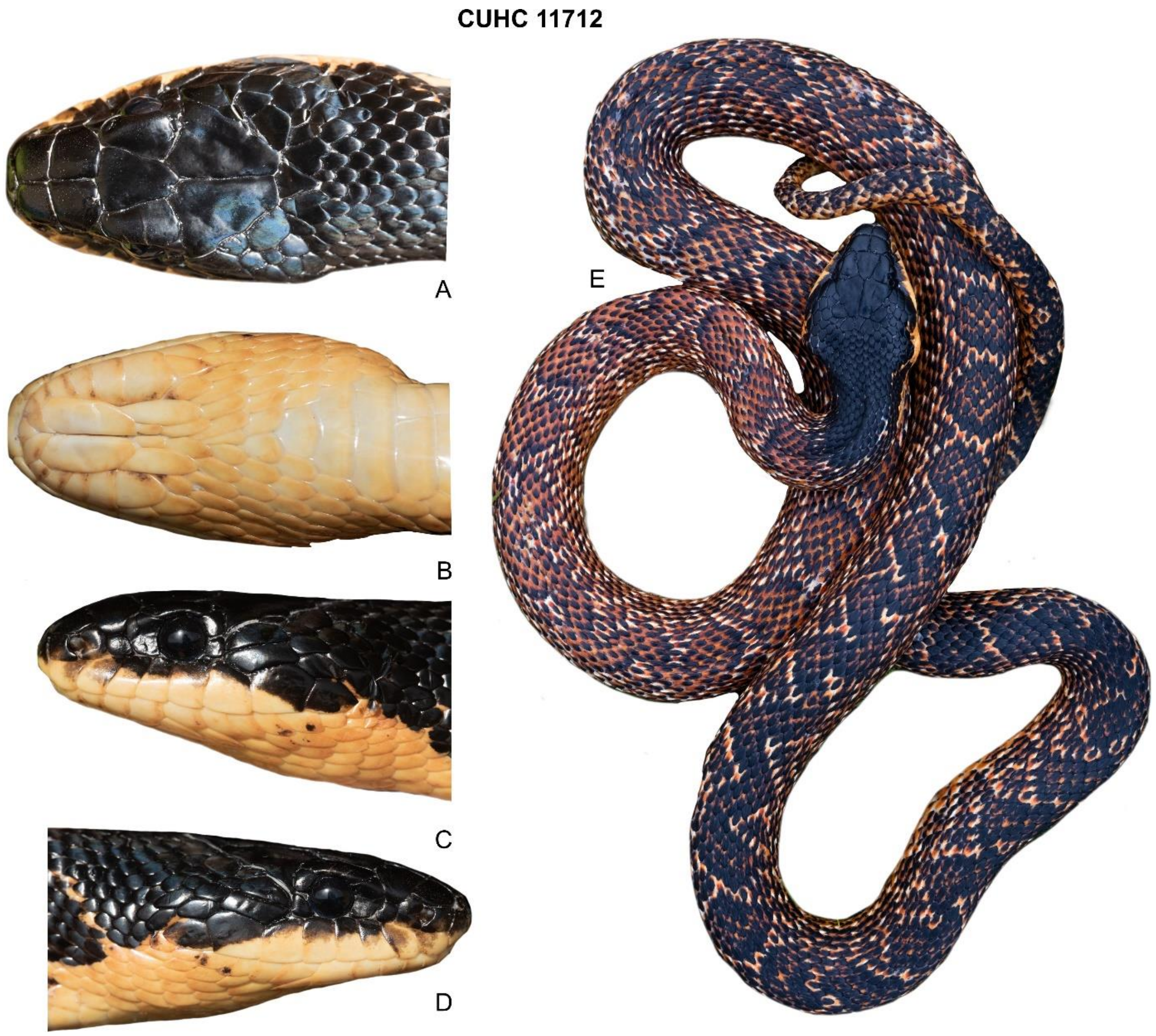

Figure S10. The adult male of *Elaphe druzei* **sp**. **nov**. in the collection of the American University of Beirut (AR-0881; AUB 1) from Barouk, Kfar Slouan, Lebanon (collected on 20 April 2006). (A-D) dorsal, ventral, and lateral views on the head, (E) dorsal and ventral (F) views on the body. Photographs by Daniel Jablonski.

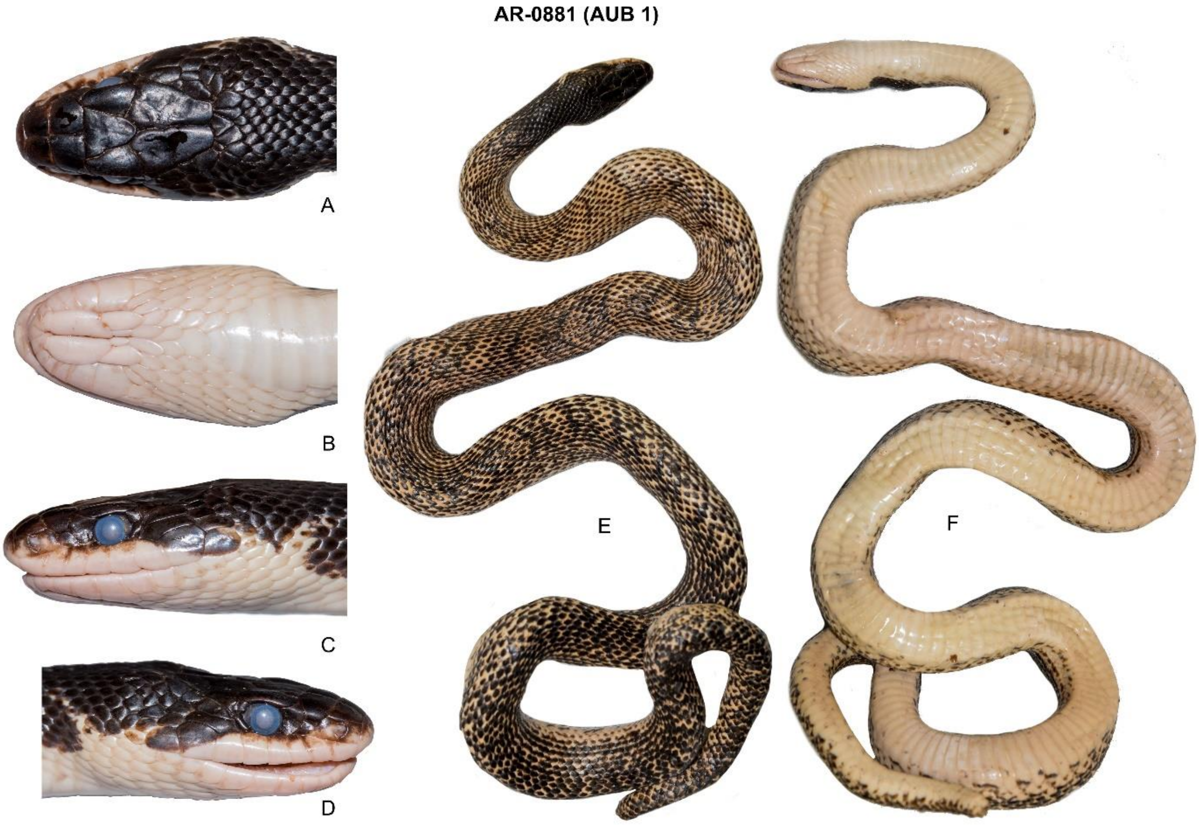

Figure S11. The adult female of *Elaphe druzei* **sp**. **nov**. in the collection of the American University of Beirut (AUB 2) from Barouk, Kfar Slouan, Lebanon. (A-D) dorsal, ventral, and lateral views on the head, (E) dorsal views on the body. Photographs by Daniel Jablonski.

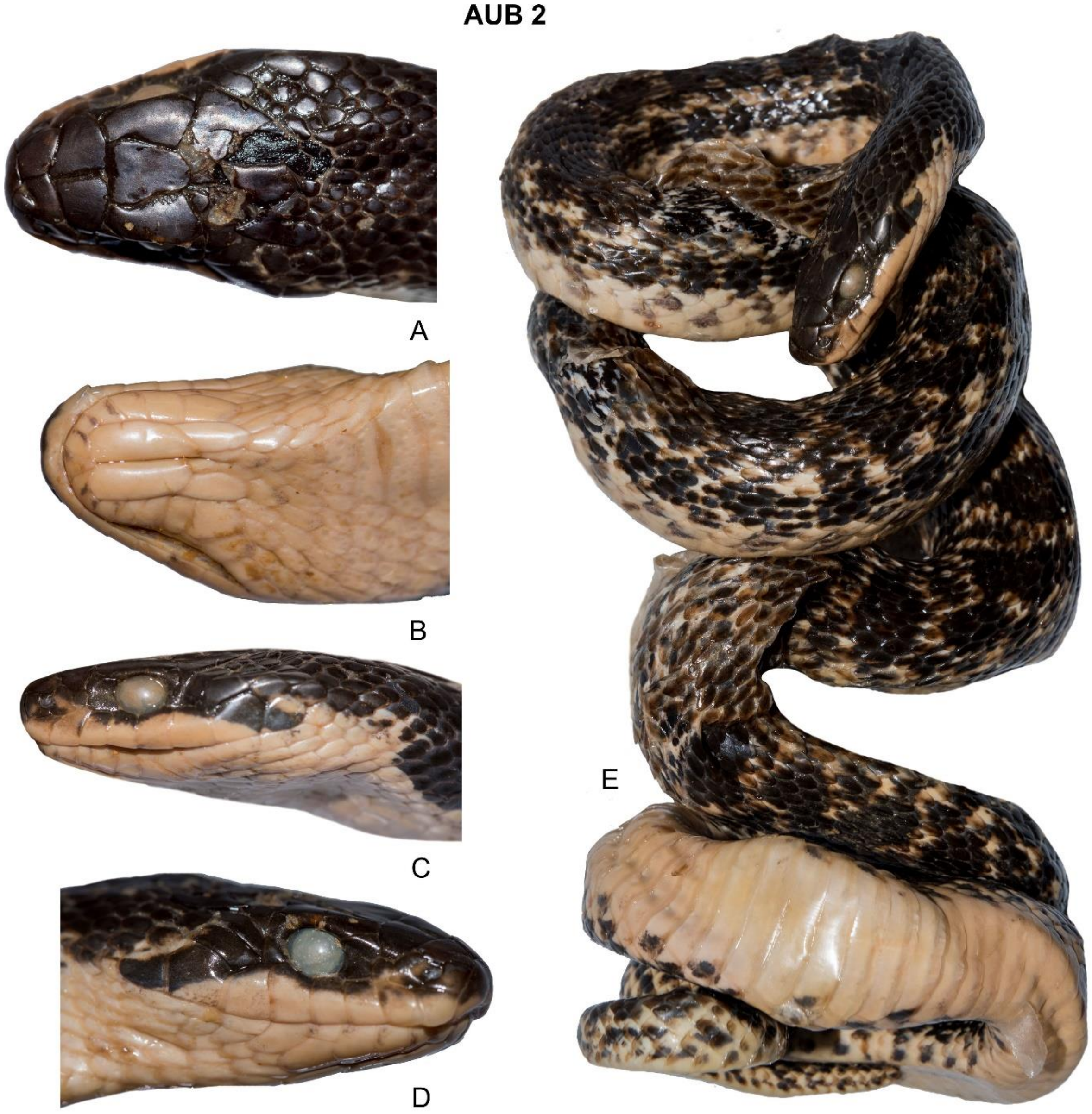

Figure S12. (A) dorsal and (B) lateral views of the head and dorsal (C) and ventral (D) views on the body of juvenile specimen of *Elaphe druzei* **sp**. **nov**. TAU-R 19144 from Hermon, 1 km southeast to the ski site, Israel (Israeli-controlled Golan Heights) (11 June 1976). Bar = 1 cm. Photographs by Marco Antônio Ribeiro-Júnior.

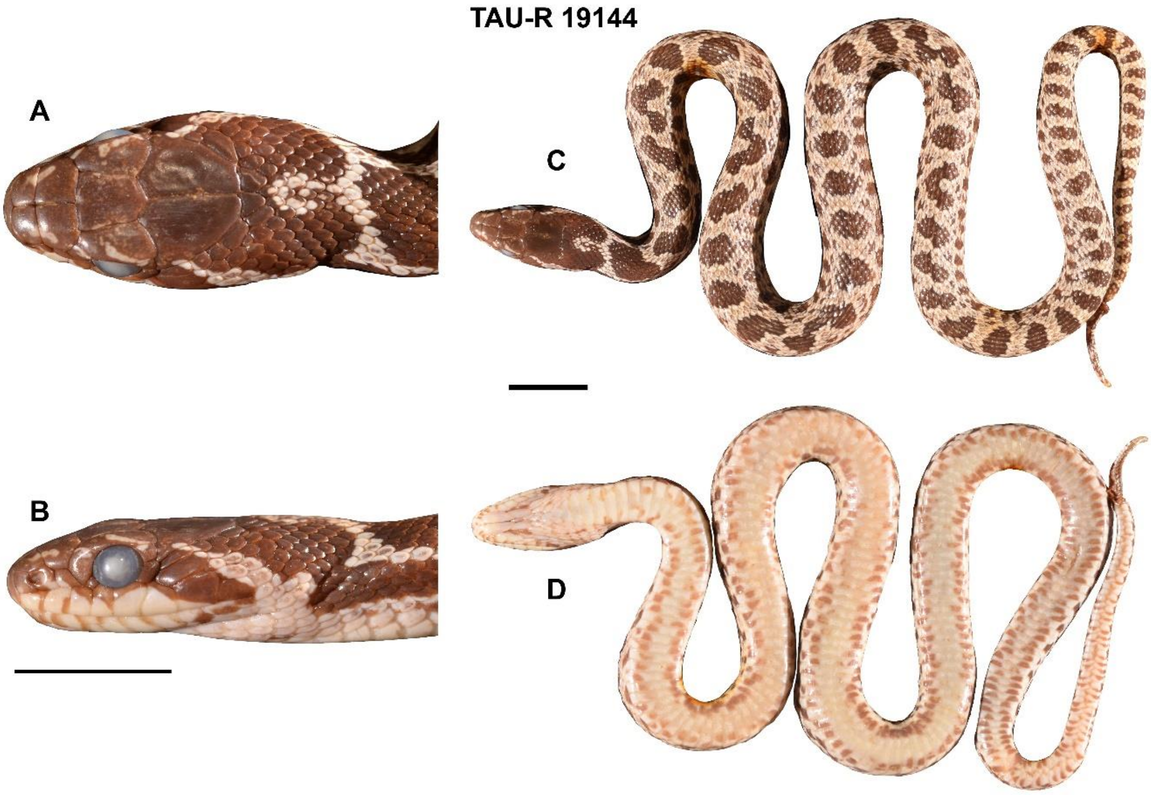

Figure S13. The adult female of *Elaphe druzei* **sp**. **nov**. from Quneitra, Camp Fauar, Golan Heights, Syria (NMW 23472). (A-C) dorsal, ventral, and lateral views on the head, (E) dorsal and ventral (F) views on the body. Bar = 1 cm. Photographs by Alice Schumacher and Daniel Jablonski.

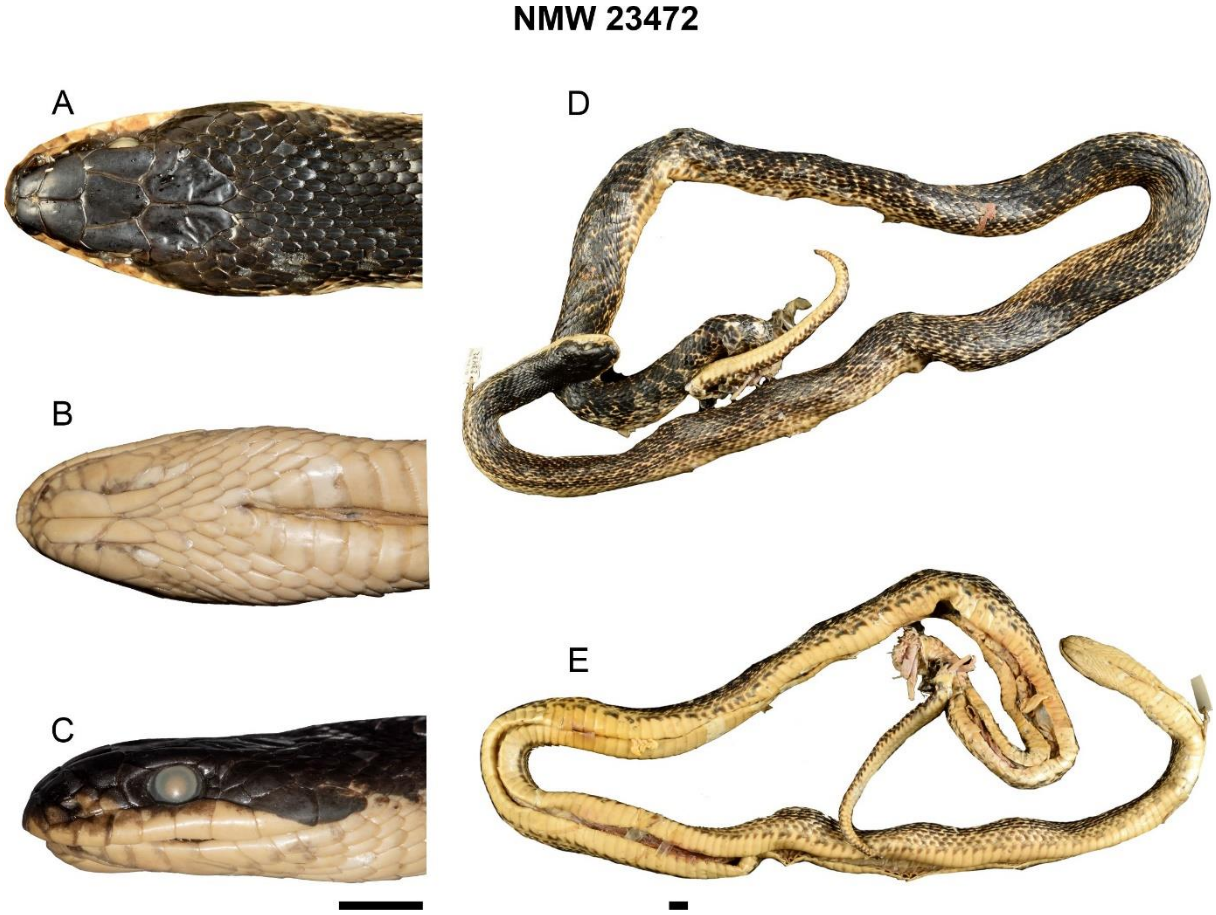

Figure S14. Possible mimicry at the dorsal color pattern of *Elaphe druzei* **sp**. **nov**. from Lebanon (A) reminiscent colour pattern of the sympatric snake species from the Levant, *Daboia palestinae* (Israel; B). Photographs by Daniel Jablonski (A) and Matthieu Berroneau (B).

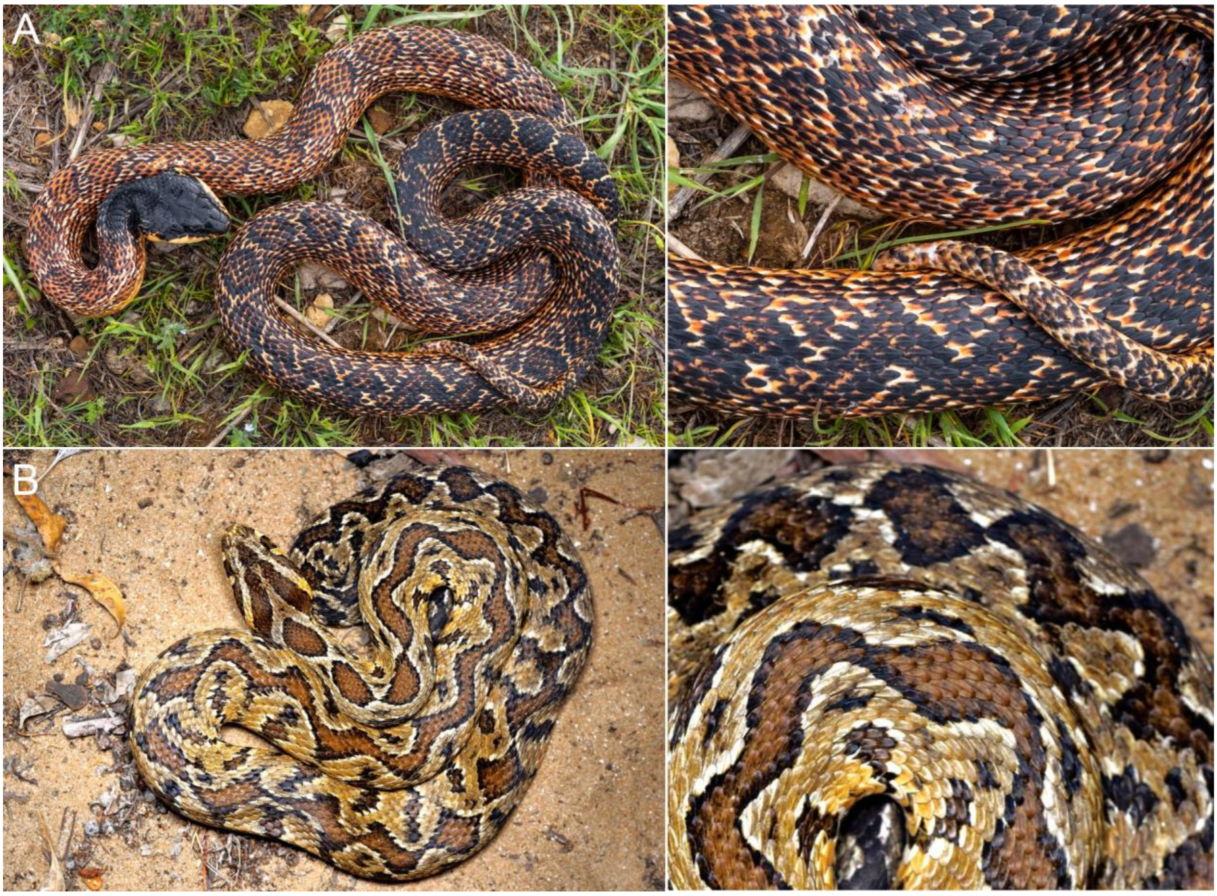

Figure S15. (A) The genus *Elaphe* as an example of use for religious, medical, or cultural purposes. The festival of the snake catchers (snake-charmers; Festa dei Serpari di Cocullo) in Cocullo, Italy in honour of St. Dominic patron showing *Elaphe quatuorlineata* that should protect people against snakebite [9-12] (photography by Matteo di Nicola). (B) A monument of the bronze snake (*Nehushtan*) on Mount Nebo, Jordan represents snake symbolism in the Levant (photography by Daniel Jablonski). *Nehushtan* is also known as a “serpent on the pole” or “bronze serpent” and may have been used in temples as a minor god of snakebite cure (originally, it should protect and cure Israelites from the bites of “fiery snakes”, probably *Echis coloratus* [13, 14]). It is not clear what snake species could *Nehushtan* represents, but the analogy with snake symbolism (*Elaphe*) used by ancient Greeks (Asclepius) or other ancient civilizations is remarkable.

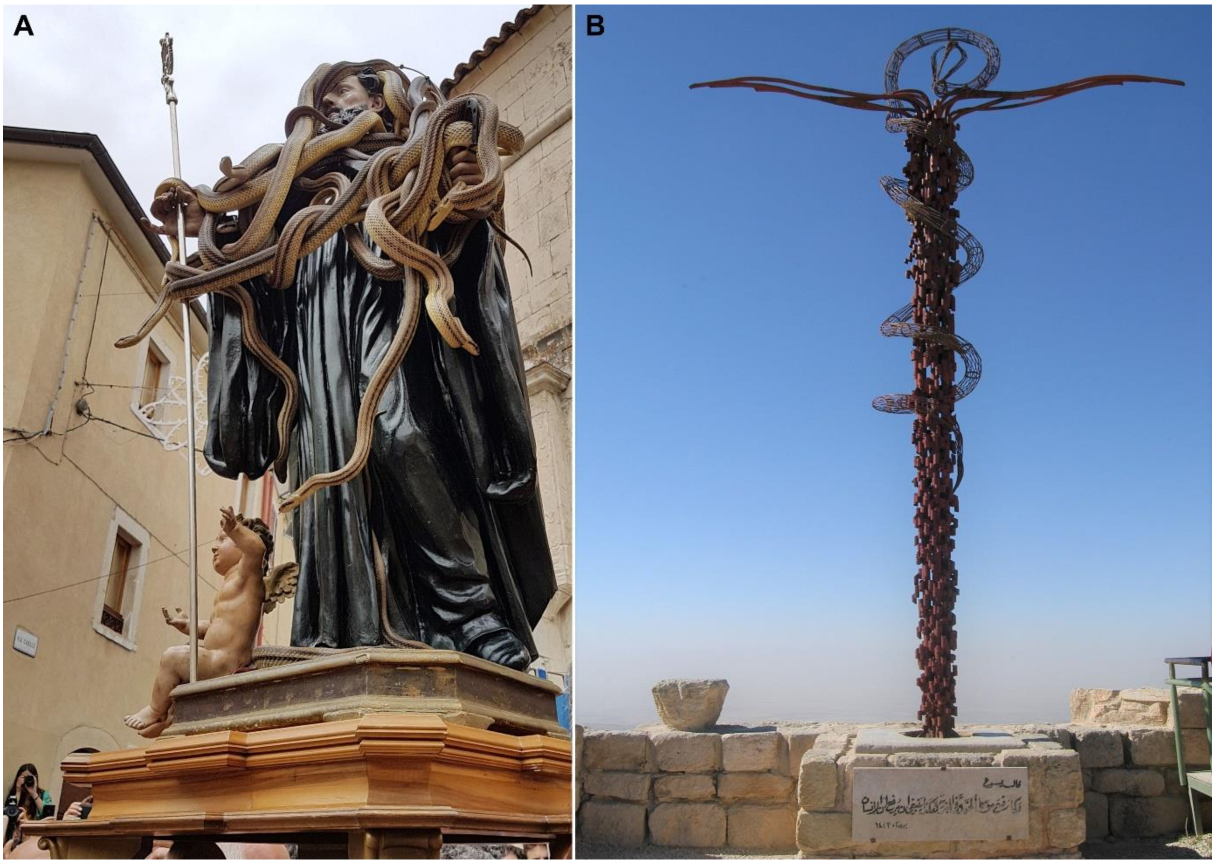

References

1. Zinner, H. Contributions to the herpetofauna of Mount Hermon No. III *Elaphe quatuorlineata* (Ophidia: Colubridae). *Israel J. Zool*. **21**, 123-127 (1972).
2. Werner, Y. L. Reptile life in the Land of Israel. 494 (Edition Chimaira, Frankfurt am Main, 2016).
3. In den Bosch, H. A. J. Die Eidechsen des Mount Hermon im Biotop mit ersten Daten zur Fortpflanzung. *Elaphe* **7**: 68-75 (1999).
4. Hraoui-Bloquet, S., Sadek, R. A., Sindaco, R. & Venchi, A. The herpetofauna of Lebanon: new data on distribution. *Zool. Middle East* **27**, 35-46 (2002).
5. In den Bosch, H. A. J, Bischoff, W. & Schmidtler, J. F. Bemerkenswerte Reptilienfunde im Libanon. *Herpetofauna* **20**, 19-32 (1998).
6. Tiedemann, F. & Häupl, M. Ein weiterer Nachweis von *Elaphe quatuorlineata sauromates* aus Syrien (Reptilia: Serpentes: Colubridae). *Salamandra* **14**, 212-214 (1978).
7. Esterbauer, H. Die Herpetofauna des östlichen Golan- und Hermongebietes Funde und Bemerkungen zur Systematik und Ökologie. *Zool. Middle East* **7**, 21-54 (1992).
8. Qawi, A., Khalil, N. & Amr, Z. S. An additional locality record of the Blotched Rat snake, *Elaphe sauromates* (Pallas, 1814) (Reptilia: Colubridae) in Syria. *Jordan J. Nat. Hist.* **6**, 50-53 (2019).
9. Bodson, L. Les Grecs et leurs serpents. Premiers résultats de taxonomique des sources anciennes. *L'antiquité classique* **50**, 57-78 (1981).
10. Angeletti, L. R., Agrimi, U., Curia, C., French, D. & Mariani-Costantini, R. Healing rituals and sacred serpents. *The Lancet* **340**, 223-225 (1992).
11. Cimatti, E. Sacred snakes of the Mediterranean. *Reptilia* **26**, 52-58 (2003).
12. Antoniou, S. A., Antoniou, G. A., Learney, R., Grandearth, F. A., Antoniou, A. I. The Rod and the Serpent: History’s Ultimate Healing Symbol. *World J. Surg*. **35**, 217-221 (2011).
13. Millett, R. P. & Pratt, J. P. Why fiery flying serpent symbolized Christ? Meridian Magazine (9 June 2000; https://www.johnpratt.com/items/docs/lds/meridian/2000/serpent.html).
14. Amzallag, N. The origin and evolution of the Saraph symbol. *Antiguo Oriente* **13**, 99-126 (2015).
